# Characterization of virulence determinants and phylogenetic background of multiple and extensively drug resistant *Escherichia coli* isolated from different clinical sources in Egypt

**DOI:** 10.1007/s00253-021-11740-x

**Published:** 2022-01-20

**Authors:** Rana El-baz, Heba Shehta Said, Eman Salama Abdelmegeed, Rasha Barwa

**Affiliations:** Department of Microbiology and Immunology, Faculty of Pharmacy, Mansoura University, Mansoura, 35516 Egypt

**Keywords:** Escherichia coli, Clermont’s phylogenetic typing, ERIC-PCR genotyping, Virulence determinants, Virulence profile, Virulence score, Resistance score, Biofilm formation, MDR, XDR

## Abstract

**Abstract:**

*Escherichia coli* is a multifaceted microbe since some are commensals, normally inhabiting the gut of both humans and animals while others are pathogenic responsible for a wide range of intestinal and extra-intestinal infections. It is one of the leading causes of septicemia, neonatal meningitis, urinary tract infections (UTIs), cystitis, pyelonephritis, and traveler’s diarrhea. The present study aims to survey the distribution and unravel the association of phylotypes, virulence determinants, and antimicrobial resistance of *E. coli* isolated from different clinical sources in Mansoura hospitals, Egypt. One hundred and fifty *E. coli* isolates were collected from different clinical sources. Antimicrobial resistance profile, virulence determinants, and virulence encoding genes were detected. Moreover, phylogenetic and molecular typing using ERIC-PCR analysis was performed. Our results have revealed that phylogroup B2 (26.67%) with the greatest content in virulence traits was the most prevalent phylogenetic group. Different virulence profiles and varying incidence of virulence determinants were detected among tested isolates. High rates of resistance to different categories of antimicrobial agents, dramatic increase of MDR (92.67%), and emergence of XDR (4%) were detected. ERIC-PCR analysis revealed great diversity among tested isolates. There was no clustering of isolates according to resistance, virulence patterns, or phylotypes. Our research has demonstrated significant phylogenetic diversity of *E. coli* isolated from different clinical sources in Mansoura hospitals, Dakahlia governorate, Egypt. *E. coli* isolates are equipped with various virulence factors which contribute to their pathogenesis in human. The elevated rates of antimicrobial resistance and emergence of MDR and XDR mirror the trend detected globally in recent years.

**Key points:**

*• Clinical E. coli isolates exhibited substantial molecular and phylogenetic diversity.*

*• Elevated rates of antimicrobial resistance and emergence of XDR in pathogenic E. coli.*

*• B2 Phylogroup with the highest VS was the most prevalent among pathogenic E. coli.*

**Supplementary Information:**

The online version contains supplementary material available at 10.1007/s00253-021-11740-x.

## Introduction

*Escherichia coli* is a ubiquitous organism having a remarkable adaptive competence in distinct ecological habitats. It is a commensal resident of the gastrointestinal tracts of several animal species and healthy humans (Rojas-Lopez et al. 2018). However, it can also cause a broad range of illnesses, ranging from enteric disease, to extra-intestinal and systemic infections (ExPEC) in humans and animals (Um et al. 2018). It has been suggested that pathogenic strains of *E. coli* have originated from commensal ones by the acquisition of chromosomal or extra-chromosomal virulence encoding genes and operons (Sobhy et al. 2020). Diarrheagenic *E. coli* (DEC) are classified, based on their pathogenicity profiles, into six pathotypes: enteropathogenic (EPEC), enterotoxigenic (ETEC), enterohamerrhagic (EHEC), enteroaggregative (EAEC), enteroinvasive (EIEC), and diffusely adherent *E. coli* (DAEC) (Ori et al. 2018). Moreover, *E. coli* is one of the commanding bacterial species leading to urinary tract infections (UTIs) including cystitis and pyelonephritis, septicemia (SEPEC), and neonatal meningitis (NMEC) (Nojoomi and Ghasemian 2019).

*E. coli* has a clonal genetic organization with a marginal level of recombination. Depending on three genetic markers (*chuA, yjaA,* and, DNA fragment TSPE4.C2), strains of *E. coli* were principally alienated into four phylogenetic groups (A, B1, B2, and D) (Clermont et al. 2000). Escobar-Páramo and coauthors further segregated these phylogenetic groups into subgroups including A0, A1, B1, B2_2_, B2_3_, D1, and D2 (Escobar-Páramo et al. 2004). However, recent phylogenetic classification has verified that *E. coli* strains might be categorized into eight phylogenetic groups: four major phylogenetic groups (A, B1, B2, and D phylotypes) and four recent groups (C, E, F, and clade I) (Clermont et al. 2013). Previous studies have indicated that commensal strains resided in phylogenetic groups A and B1, while the extra-intestinal strains fall into phylogenetic groups B2 and D (Kaper 2005; Mosquito et al. 2015). Therefore, phylogenetic clustering of *E. coli* strains is valuable not only to portray *E. coli* communities but also to depict the association between phylotypes and diseases caused by the organism as well.

Pathogenic *E. coli* has an array of virulence determinants that play a significant role in its pathogenesis. Moreover, it has been revealed that pathogenic strains have a superior prevalence of virulence traits than commensal ones (Sobhy et al. 2020). These virulence determinants include structural factors (fimbriae and flagella), iron-acquisition systems, secreted toxins, and capsules, which evade the host defenses, invade host tissues, and ultimately elicit local inflammation in the host (El-Shaer et al. 2018). Fimbrial adhesins including Type 1 fimbriae, P fimbriae, S fimbriae, and other fimbrial adhesins partake in adherence and colonization of the host epithelium (Sobhy et al. 2020). To secure ample level of iron, pathogenic *E. coli* have/express redundant iron acquisition systems in response to iron limiting conditions inside the host (Rehman et al. 2017). A number of toxins are produced by different strains of *E. coli* including heat labile (LTI and LTII), heat-stable (*STa* and STb), Shiga toxins (stx1 and stx2), cytotoxic necrotizing factor (cnf1), and hemolysin toxins, which play different roles in their pathogenesis (Ochoa et al. 2016).

An array of antibiotics could be used for management of *E. coli* infections in animals and humans. Nevertheless, recent reports have indicated elevated levels of resistance to different classes of antibiotics including β-lactams, fluoroquinolones, tetracyclines and, aminoglycosides among pathogenic *E. coli* isolates worldwide and in Egypt (Dehkordi et al. 2020, Duan et al. 2020, Flament-Simon et al. 2020, Khairy et al. 2020, and Masoud et al. 2021). The increased usage of antibiotics in clinical settings has led to the evolution of multidrug resistant (MDR), extensively drug resistant (XDR), and pan-drug resistant (PDR) strains leading to failure of treatment, complications, and increased rates of morbidity and mortality (Parajuli et al. 2017). Antimicrobial resistance is more predominant among pathogenic organisms compared to the commensal ones. The correlation between resistance and virulence factors could be a result of the successive exposure of pathogenic organisms to antibiotics (Ansari et al. 2015; Rehman et al. 2017).

The present study aims to explore the dissemination and conceivable association of phenotypes, phylogenetic groups, antimicrobial resistance, and virulence traits among pathogenic *E. coli* isolates collected from different clinical sources in Mansoura, Dakahlia governorate, Egypt.

## Materials and methods

### Specimen collection

Bacterial isolates were collected from patient specimens between September 2017 and August 2018 from different hospitals in Mansoura, Dakahlia governorate, Egypt. Isolates were identified as *E. coli* based on standard laboratory protocols (Colle and Watt, 1996). *E. coli* ATCC 25,922 and *K. pneumoniae* ATCC 33,495 were used as control strains. Moreover, all isolates were screened for the *uidA* gene by polymerase chain reaction (PCR) as confirmation marker (Table 1) (Ramirez Castillo et al. 2013). All isolates were cultured in Luria–Bertani broth and preserved in 25% v/v glycerol (Colle and Watt, 1996).

**Table 1:** List of oligonucleotides used for identification, phylotyping, and virulence factors detection among *E. coli* clinical isolates

Virulence traits	Virulence gene	Nucleotide sequence (5′-3′)	Amplicon size (bp)	Annealing temp °C	Reference
**Adhesions**					
Afimbriae/Dr-binding fimbriae	*afa/dra* F	GGCAGAGGGCCGGCAACAGGC	592	62	Johnson and Stell 2000
	*afa/dra* R	CCCGTAACGCGCCAGCATCTC			
P-fimbrial usher protein	*papC* F	GACGGCTGTACTGCAGGGTGTGGCG	350	65	Blanco et al. 1997
	*papC* R	ATATCCTTTCTGCAGGGATGCAATA			
P-fimbria tip adhesin (allele II)	*papG* F	CTGTAATTACGGAAGTATTTCTG	1070	55	Johnson 1998
	*papG* R	ACTATCCGGCTCCGGATAAACCAT			
S-fimbriae	*sfaS* F	GTGGATACGACGATTACTGTG	240	63	Johnson and Stell 2000
	*sfaS* R	CCGCCAGCATTCCCTGTATTC			
The major subunit of a putative fimbriae	*yfcV* F	ACATGGAGACCACGTTCACC	292	50	Johnson and Stell 2000
	*yfcV* R	GTAATCTGGAATGTGGTCAGG			
**Toxins**					
Heat-stable enterotoxin ST-I group a	*sta1* F	TCTTTCCCCTCTTTTAGTCAG	165	52	Woodward et al. 1992
	*sta1* R	ACAGGCAGGATTACAACAAAG			
Heat-stable enterotoxin ST-I group b	*sta2* F	CGCTCAGGATGCTAAACCA	300	53	Woodward et al. 1992
	*sta2* R	AATTCACAGCAGTAATTGCT			
Heat-labile enterotoxin LT subunit A	*eltA* F	GGCGACAGATTATACCGTGC	450	52	Aranda et al. 2007
	*eltA* R	CGGTCTCTATATTCCCTGTT			
Vacuolating autotransporter toxin	*vat* F	TCAGGACACGTTCAGGCATTCAGT	1100	57	Spurbeck et al. 2012
	*vat* R	GGCCAGAACATTTGCTCCCTTGTT			
Cytotoxic necrotizing factor I	*cnf1* F	AGGATGGAGTTTCCTATGCAGGAG	498	57	Johnson and Stell 2000
	*cnf1* R	CATTCAGAGTCCTGCCCTCATTATT			
EAST1	*astA* F	GCCATCAACACAGTATATCC	106	50	Moon et al. 2005
	*astA* R	GAGTGACGGCTTTGTAGTCC			
Shiga toxin I	*stx1* F	TCCTGGTACAACTGCGGTTAC	505	60	Sallam et al. 2013
	*stx1* R	ACGCACTCTTCCATCTACCG			
Shiga toxin II	*stx2* F	CTGGCGTTAATGGAGTTCAGTGG	380	60	Sallam et al. 2013
	*stx2* R	CCTGTCGCCAGTTATCTGACA			
Intimin	*eae* F	GACCCGGCACAAGCATAAGC	384	62	Paton and Paton 1998
	*eae* R	CCACCTGCAGCAACAAGAGG			
**Serum resistance**					
Group 2 capsule system	*kpsMTII* F	GCGCATTTGCTGATACTGTTG	392	60	Johnson and Stell 2000
	*kpsMTII* R	CATCCAGACGATAAGCATGAGCA			
**Iron acquisition system**					
Ferric aerobactin receptor	*iutA* F	ATCAGAGGGACCAGCACGC	253	60	Abdelmegeed et al. 2015
	*iutA* R	TTCAGAGTCAGTTTCATGCCGT			
Ferric yersiniabactin receptor	*fyuA* F	TGATTAACCCCGCGACGGGAA	880	55	Johnson and Stell 2000
	*fyuA* R	CGCAGTAGGCACGATGTTGTA			
Heme binding protein	*chuA* F	ATGGTACCGGACGAACCAAC	288	59	Clermont et al. 2013
	*chuA R*	TGCCGCCAGTACCAAAGACA			
**Primers used for identification of *E. coli* and ERIC typing**					
Β-glucuronidase	*uidA* F	ATGTGCTGTGCCTGAACC	450	55	Ramirez Castillo et al. 2013
	*uidA* R	ATTGTTTGCCTCCCTGCTG			
ERIC- PCR	*ERIC-1R*	ATGTAAGCTCCTGGGGATTCAC	Variable	48	Versalovic et al. 1991
	*ERIC-2*	AGTAAGTGACTGGGGTGAGCG			
**Primers used in the extended quadruplex phylotyping method**					
*Quadruplex*					
*ChuA*	*chuA.1b*	ATGGTACCGGACGAACCAAC	288	59	Clermont et al. 2013
	*chuA.2*	TGCCGCCAGTACCAAAGACA			
*yjaA*	*yjaA.1b*	CAAACGTGAAGTGTCAGGAG	211	59	Clermont et al. 2013
	*yjaA.2b*	AATGCGTTCCTCAACCTGTG			
TspE4.C2	*TspE4C2.1b*	CACTATTCGTAAGGTCATCC	152	59	Clermont et al. 2013
	*TspE4C2.2b*	AGTTTATCGCTGCGGGTCGC			
*arpA*	*AceK.f*	AACGCTATTCGCCAGCTTGC	400	59	Clermont et al. 2013
	*ArpA1.r*	TCTCCCCATACCGTACGCTA			
Group E (*arpA*)	*ArpAgpE.f*	GATTCCATCTTGTCAAAATATGCC	301	57	Clermont et al. 2013
	*ArpAgpE.r*	GAAAAGAAAAAGAATTCCCAAGAG			
Group C (*trpA*)	*trpAgpC.1*	AGTTTTATGCCCAGTGCGAG	219	57	Clermont et al. 2013
	*trpAgpC.2*	TCTGCGCCGGTCACGCCC			
Internal control (*trpA*)	*trpBA.f*	CGGCGATAAAGACATCTTCAC	489	59	Clermont et al. 2013
	*trpBA.r*	GCAACGCGGCCTGGCGGAAG			

*F* Forward primer; *R* Reverse primer; bp: base pair

### Antimicrobial susceptibility testing

Antimicrobial susceptibility profile of *E. coli* isolates was carried out by Kirby-Bauer disk diffusion method on Mueller–Hinton agar media. Susceptibility to colistin was evaluated by micro-broth dilution method. The results were interpreted according to the recommendations of Clinical and Laboratory Standards Institute (CLSI), US Food and Drug Administration (FDA), and European Committee on Antimicrobial Susceptibility Testing (EUCAST) (CLSI 2018). *E. coli* ATCC 25,922 was used as a control strain. The following antibiotic disks (Oxoid, UK) were used to define resistance profiles among *E. coli* clinical isolates: Gentamicin (10 µg), Tobramycin (10 µg), Amikacin (30 µg), Netilmicin (30 µg), Ceftaroline (30 µg), Ticarcillin-Clavulanate (75/10 µg), Ertapenem (10 µg), Imipenem (10 µg), Meropenem (10 µg), Doripenem (10 µg), Cefazolin (30 µg), Cefuroxime (30 µg), Cefotaxime (30 µg), Ceftriaxone (30 µg), Ceftazidime (30 µg), Cefepime (30 µg), Cefoxatin (30 µg), Ciprofloxacine (5 µg), Trimethoprim-Sulphamethoxazole (1.25/23.75 µg), Tigecycline (15 µg), Aztreonam (30 µg), Ampicillin (10 µg), Amoxicillin-Clavulanic acid (20/10 µg), Ampicillin-Sulbactam (10/10 µg), Chloramphenicol (30 µg), Fosfomycin (200 µg), Colistin-sulfate (100 µg, Sigma-Aldrich, Germany), Tetracycline (30 µg), Doxycycline (30 µg), and Minocycline (30 µg).

Bacterial isolates were classified according to Margiorakos and coauthors into MDR, XDR, and PDR. MDR was identified as non-susceptibility to at least one antibiotic in three or more antimicrobial groups while XDR was non-susceptibility to at least one antibiotic in all except for two or fewer antimicrobial groups. PDR was defined as resistance to all the present categories of antibiotics available for therapeutic treatment (Magiorakos et al. 2012). The resistance score (RS) was identified as the number of antimicrobial categories to which resistance was detected (Johnson et al. 2016).

### Phenotypic detection of virulence determinants of E. coli clinical isolates

#### Biofilm assay by tissue culture plate method

Biofilm formation was assessed quantitatively using polystyrene 96-well microtiter plate method as described previously to evaluate the attachment and capacity of biofilm formation under static conditions (Schönborn et al. 2017; Singh et al. 2017)**.** *E. coli* isolates were grown in tryptic soy broth (TSB) complemented with 0.25% anhydrous glucose at 37 °C for 24 h. The optical density (OD) of inocula was adjusted to approximately equal to 10^8^ CFU/ml (0.2–0.257 at 600 nm using spectrophotometer). Two hundred microliters of pre-adjusted culture was inoculated in four adjacent wells. In the same plate, a negative control containing tryptic soy broth only was performed. After overnight incubation of the plate without shaking at 37 °C, the content of every well was aspirated and washed by 200 μl of phosphate-buffered saline (PBS, pH 7.4) three times with shaking to get rid of any non-adherent cells. Fixation of the adherent cells was performed by the addition of 150 μl of absolute methanol to each well, incubated for 15 min, then left to dry. The fixed biofilm was then stained using 150 μl (1% w/v) crystal violet for 20 min. Following staining, the plate was rinsed three times with distilled water then left to dry. The bound dye with biofilm was re-solubilized using 150 μl (33% v/v) acetic acid per well then the OD was measured at 570 nm. The mean of the four ODs for each isolate was calculated. To assess the capacity of biofilm formation of *E. coli* isolates, optical density cut-off value (ODc) was set as three standard deviations over the mean OD of the negative control. Isolates were categorized as follows: non-biofilm producer, NP (OD ≤ ODc); weak biofilm producer, WP (ODc < OD_WP_ ≤ 2 × ODc); moderate biofilm producer, MP (2 × ODc < OD_MP_ ≤ 4 × ODc), and strong biofilm producer, SP (OD_SP_ > 4 × ODc) (Schönborn et al. 2017; Singh et al. 2017).

#### Serum resistance assay

All clinical isolates were analyzed for serum resistance using turbidimetric assay as described previously (Vandekerchove et al. 2005). In a 96-well microtiter plate, 50 μl of bacterial culture of OD_600_ 0.1 was mixed with 150 µl of normal human serum. Using a microplate reader, the initial and final absorbance (after 3 h of incubation at 37 °C) were determined at 620 nm. Absorbance of each isolate was calculated as the mean of three replicates, and then the percentage of remaining absorbance in relation to the initial absorbance was determined. If the remaining absorbance was greater than 150% of the initial absorbance, isolate was considered serum resistant (SR). If it ranged between 125 and 150%, isolate was assigned intermediate serum resistant (IR); between 100 and 125%, it was considered slow-intermediate serum resistant (SIR); and less than 100% was designated serum sensitive (S) (Vandekerchove et al. 2005). *E. coli* strain BL21 (DE3) was used as a negative control (serum sensitive) (Vandekerchove et al. 2005).

#### Swimming motility assay

Motility assay of *E. coli* isolates was performed according to the previously described protocols (Murinda et al. 2002). Briefly, the bacterial culture was stabbed into motility test medium with triphenyltetrazolium chloride (TTC). After incubation for 24–48 h at 37 °C, growth was indicated by the presence of the red color; and as motility occurs, small to very large regions of color could be observed around the area of inoculation.

#### Screening of hemolysin activity

The hemolytic activity of *E. coli* isolates was assessed by streaking isolates onto 5% sheep blood agar plates. After incubation at 37 °C for 24 h, plates were inspected for signs of α-hemolysis (a green-hued zone around colonies), β-hemolysis (clear zones around colonies), or γ-hemolysis (no halo around colonies) (Maragkoudakis et al. 2006). Moreover, screening of hemolysin activity was also determined by tube assay method as described previously (Rossignol et al. 2008). *E. coli* isolates were grown in tryptic soy broth at 37 °C for 48 h with shaking. Bacterial extract was obtained by centrifugation at 10,000 rpm for 10 min. Aliquots of 500 µl RBCs suspension (2% O-type RBCs in 10 mm Tris HCl, pH 7.4) and 500 µl of bacterial extract were incubated in a heat block at 37 °C for 2 h. Positive (T) and negative (B) controls were done by treating the RBCs with 0.1% sodium dodecyl sulfate (SDS) and distilled water, respectively. The amount of hemoglobin released for each sample (X) was measured at 540 nm after centrifugation at 10,000 rpm for 10 min. The percentage (%) of lysed cells was calculated as follows: % = ((X-B)/(T-B)) × 100. The experiment was repeated three times (Rossignol et al. 2008).

#### DNA extraction

A single colony from overnight growth of *E. coli* test isolate on LB agar plates was suspended in 100 μl sterile distilled water and heated at 95 °C for 10 min in a heat block. Cell lysates were centrifuged at 10,000 rpm for 5 min, and the supernatants were separated, stored at − 20 °C, and used as templates for polymerase chain reaction (PCR) (Said et al. 2018).

#### Molecular detection of E. coli virulence genes

The prevalence of virulence genes among *E. coli* isolates was screened by PCR targeting adhesins: Afimbriae/Dr-binding fimbriae (*afa/dra)* (Johnson and Stell 2000), P-fimbrial Usher Protein (*papC) (*Blanco et al. 1997*)*, P-fimbria tip adhesin (*papG)* (Johnson 1998), The major subunit of a putative fimbriae (*yfcV)* (Johnson and Stell 2000), S fimbriae (*sfaS)* (Johnson and Stell 2000); iron acquisition systems: Ferric yersiniabactin receptor (*fyuA)* (Johnson and Stell 2000), Ferric aerobactin receptor (*iutA)* (Abdelmegeed et al. 2015), Heme binding protein (*chuA)* (Clermont et al. 2013); Group 2 capsule system (*kpsMTII)* (Johnson and Stell 2000); toxins: Heat-stable enterotoxins (*sta1* and *sta2)* (Woodward et al. 1992), Heat-labile enterotoxin (*eltA)* (Aranda et al. 2007), EAST1 (*astA*) (Moon et al. 2005), Shiga toxin I (*stx1)* (Sallam et al. 2013), Shiga toxin II (*stx2)* (Sallam et al. 2013), Cytotoxic necrotizing factor I *(cnf1)* (Johnson and Stell 2000), Vacuolating autotransporter toxin (*vat)* (Spurbeck et al. 2012), and Intimin *(eae)* (Paton and Paton 1998). Primer pairs (InvitrogenTM, UK) used are listed in Table 1. Polymerase chain reaction amplifications were performed in a DNA thermocycler with a programmed cycling conditions: initial denaturation at 94 °C for 5 min followed by 30 cycles each composed of denaturation at 94 °C for 40 s, annealing at the specified temperature (Table 1) for 40 s and extension at 72 °C for 1 min, then final extension at 72 °C for 10 min. For visualization of the amplicons, agarose gel electrophoresis was performed and compared with appropriate DNA markers: GeneRuler 100 bp or GeneRuler 100 bp plus (ThermoFisher Scientific^Tm^, UK). The virulence gene score was defined as the number of virulence genes/operons identified, adjusted for multiple detection of *sfa* or *foc*, *pap,* and *kpsM II* operons (Johnson et al. 2017).

#### Clermont’s phylogenetic typing

*E. coli* isolates were subjected to phylotyping as described previously (Clermont et al. 2013). The reaction was performed on a thermal cycler with a programmed cycling conditions: initial denaturation at 94 °C for 5 min and 35 cycles of denaturation at 94 °C for 30 s, annealing at 59 °C for 30 s (quadruplex) or at 57 °C for 30 s (group C and E), extension at 72 °C for 1 min, and eventually final extension at 72 °C for 7 min (Table 1). Amplicons were electrophoresed using 1.2% agarose gel in Tris–borate-EDTA (TBE) buffer, visualized using ethidium bromide staining, and photographed under UV light. *E. coli* isolates were assigned to distinct phylotypes: A, B1, B2, C, D, E, F, and Clade I (Clermont et al. 2013). For superior strain-level designation, the subgroups/phylotypes were determined (Escobar-Páramo et al. 2004).

#### Molecular typing by enterobacterial repetitive intergenic consensus PCR (ERIC-PCR) analysis

Molecular genotyping of *E. coli* clinical isolates was performed by (ERIC-PCR) using specific primers (Table 1) (Versalovic et al. 1991). Amplification was carried out with the following conditions: initial denaturing at 94 °C for 5 min, 35 cycles of denaturation at 94 °C for 40 s, annealing at 48 °C for 1 min, and extension at 72 °C for 1.5 min, then the reaction was terminated by a final extension of 72 °C for 10 min. The amplified DNA fragments were separated using 2% agarose gel and the resulting patterns obtained were scanned and evaluated using GelJ software (Aladarose et al. 2019; Heras et al. 2015). Similarity matrix, based on Dice’s coefficient, was calculated and the corresponding dendrogram was constructed using the unweighted pair-group method with arithmetic averages (UPGMA) (Saitou and Nei 1987).

#### Statistical analysis

Comparisons of frequencies of dispersion of genotypic and phenotypic features between *E. coli* isolated from different clinical sources were assessed using the chi-square test or Fisher’s exact test (*P* < 0.05). Moreover, correspondence analysis (CA) and multiple correspondence analysis (MCA) were performed using R (version 4.1.2) to explore the structure of the data. The association between biofilm formation capacity and virulence traits was assessed using univariable logistic regression followed by multivariable logistic regression analysis, where OR with 95% CI was reported and *P*-value < 0.05 was considered statistically significant. Moreover, Kruskal–Wallis test was used to compare virulence and resistance scores (VS and RS, respectively) among different phylotypes followed by Mann–Whitney *U* test to compare each pair of groups. The *P*-value was adjusted for multiple comparisons using Bonferroni correction method. All data were evaluated using SPSS software (version 20.0; SPSS, Chicago, IL, USA).

## Results

### Isolation and identification of E. coli from different clinical sources

Over the period from September 2017 to August 2018, 332 clinical specimens were collected from various hospitals in Mansoura, Dakahlia governorate, Egypt. Patient’s age ranged from 10 to 65 years old. One hundred and fifty non-duplicate isolates (one isolate/patient) were identified as *E. coli* based on microscopic examination and standard laboratory protocols including indole, methyl red (MR), Voges-Proskauer (VP), citrate utilization, and green metallic sheen on eosin methylene blue medium. The *uidA* gene was profitably amplified in all *E. coli* isolates confirming its identification. *E. coli* isolates were recovered from different clinical sources including urine (*n* = 69), wounds (*n* = 40), blood (*n* = 6), stool (*n* = 18), urinary bladder drain (*n* = 9), sputum (*n* = 5), throat swab (*n* = 2), and vaginal smear (*n* = 1) (Table S1).

### Phylogenetic distribution of E. coli clinical isolates

**Fig. 1:**
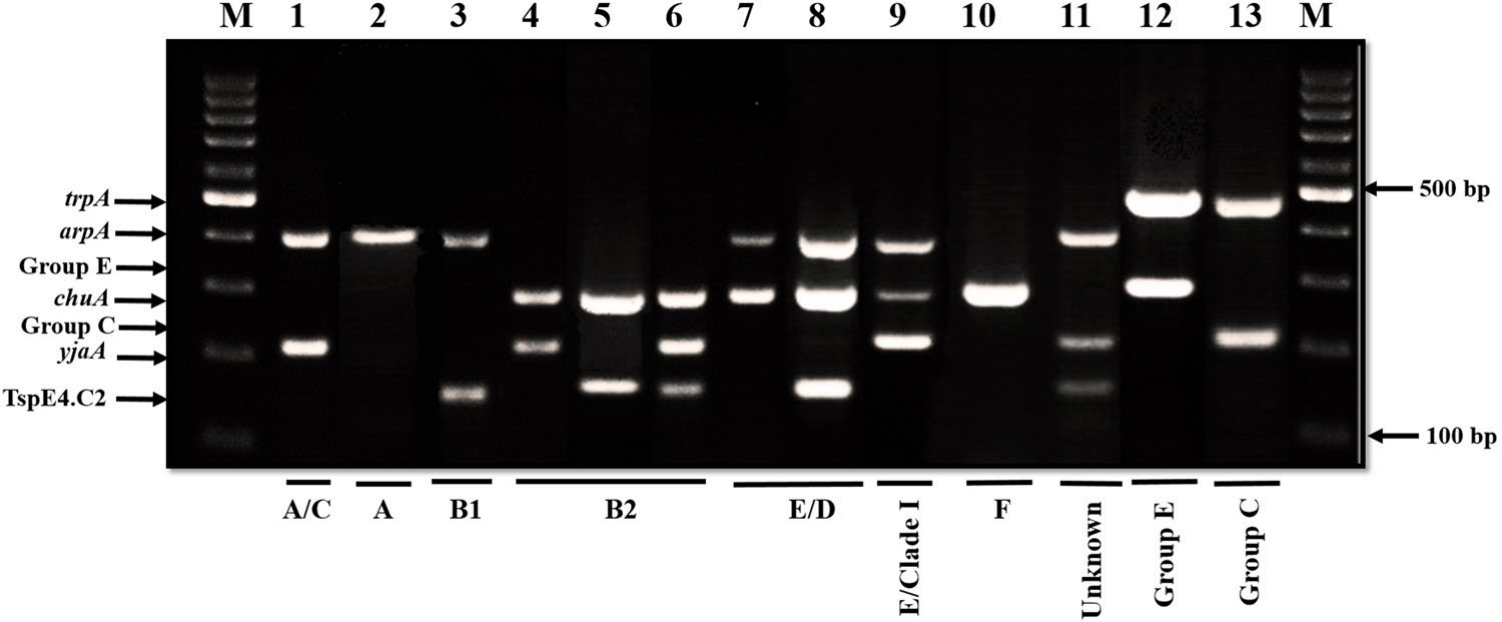
Agarose gel electrophoresis for Quadruplex PCR profiles of *E. coli* clinical isolates according to new Clermont Phylotyping method. Lane M is the molecular weight marker

Phylogenetic investigation, based on Clermont’s phylogenetic typing, showed great diversity among *E. coli* clinical isolates. All eight phylotypes were detected among *E. coli* clinical isolates (Table S1, Fig. 1). Forty isolates (26.67%) were assigned to phylotype B2, where 34 isolates were sub-grouped to B2_3_ and 6 isolates to B2_2_, followed by 38 isolates (25.33%) assigned to B1. Fifteen isolates were assigned to A phylotype, where 12 isolates were sub-grouped to A1 and 3 isolates to A0. Twelve isolates were assigned to D phylotype, where 6 isolates were sub-grouped to D1 and 6 isolates to D2, while, 15, 17, 7, and 5 isolates were assigned to C, E, F, and Clade I phylotypes, respectively. Only one isolate could not be assigned to any phylogenetic group and considered unknown. Statistical analysis has revealed correlation between the phylotypes of isolates and their clinical source (*P* < 0.05, Table S2 .A and Fig. S2).

### Genotyping by using ERIC analysis

**Fig. 2:**
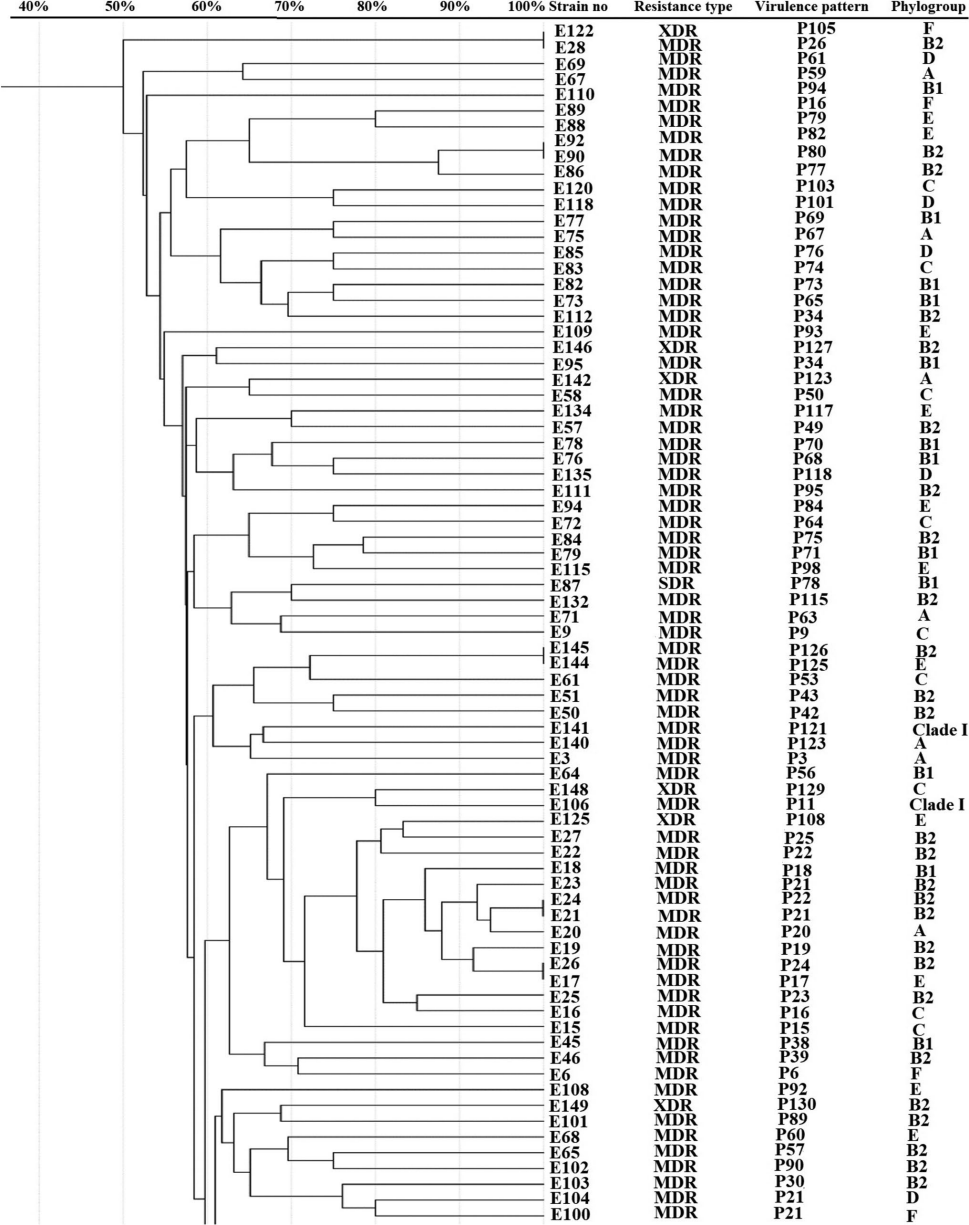

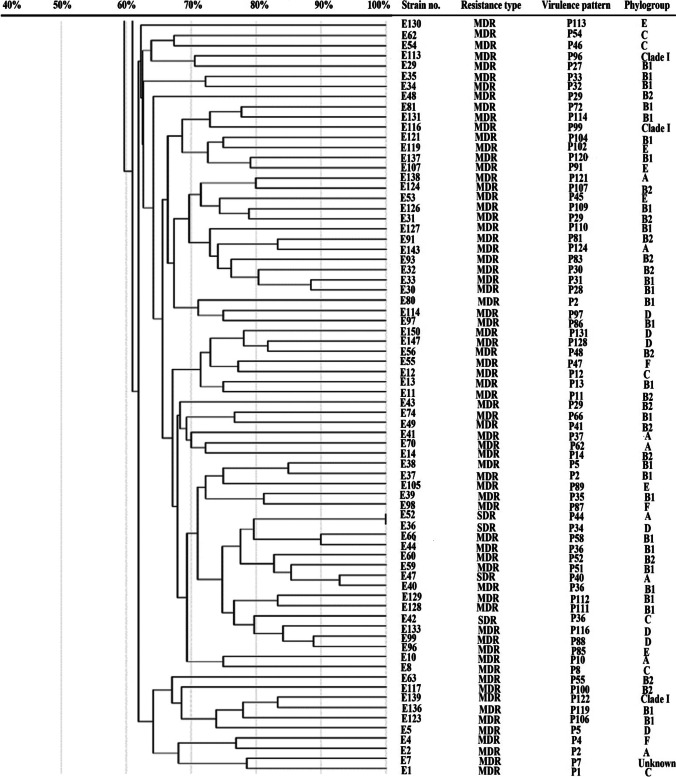
Molecular typing of *E. coli* isolates collected from different clinical sources using ERIC-PCR analysis. Dendogram representing *E. coli* ERIC-PCR patterns, resistance and virulence patterns, and phylogroups. Similarity of clustering analysis was performed using UPGMA and Jaccard coefficient at 80% similarity

The relationship between *E. coli* clinical isolates was analyzed by using ERIC-PCR analysis using GelJ software and UPGMA clustering analysis (Fig. 2). ERIC-PCR method revealed great diversity among the tested isolates. There was no clustering of the isolates based on phylotyping, antibiotic resistance profile, virulence factors, or virulence profiles.

### Molecular detection of virulence genes among E. coli clinical isolates

The prevalence of virulence genes among *E. coli* isolates was screened by PCR. Among adhesins, Afimbriae/Dr-binding fimbriae (*afa/dra)* was detected in 21 isolates (14%), S-fimbriae (*sfaS)* in 91 isolates (60.67%), The major subunit of a putative fimbriae (*yfcV)* in 50 isolates (33.33%), and P-fimbrial Usher Protein (*papC)* in 74 isolates (49.33%), while P-fimbria tip adhesin (*papG) allele II* in 32 isolates (21.33%). Iron acquisition systems were detected in *E. coli* clinical isolates, Ferric yersiniabactin receptor (*fyuA*) was successfully amplified in 85 isolates (56.67%), and Ferric aerobactin receptor (*iutA)* in 52 isolates (34.67%), while Heme binding protein (*chuA)* in 81 isolates (54%). Group 2 capsule system (*kpsMTII)* was detected in 104 isolates (69.33%). An array of toxins was detected in *E. coli* clinical isolates including Heat-stable enterotoxins ST-I group b (*sta2)* in 23 isolates (15.33%), EAST1 (*astA*) in 61 isolates (40.67%), Shiga toxin II (*stx2)* in 85 isolates (56.67%), Cytotoxic necrotizing factor I *(cnf1)* in 63 isolates (42%), Vacuolating autotransporter toxin (*vat)* in 10 isolates (6.67%), and Intimin *(eae)* in 23 isolates (15.33%). None of the isolates harbored Heat-stable enterotoxins ST-I group a (*sta1*), Heat-labile enterotoxin (*eltA)*, or Shiga toxin I (*stx1)* (Fig. S1).

The profile of virulence determinants was extremely diverse where up to 12 genes (VS 0 to 12) were detected among the tested *E. coli* clinical isolates (Table S1) and 131 unique virulence profiles were detected (Fig. 2). Moreover, statistical analysis has indicated significant association between phylotypes and virulence scores (VS, Table S3.A). Among different phylotypes, B2 isolates demonstrated the highest VS which was significantly higher than that of A, B1, and C phylotypes (Table S3.B).

### Phenotypic detection of virulence determinants of E. coli clinical isolates

#### Phenotypic screening of motility, hemolysin, and serum resistance

Hemolysin activity (β-hemolysis) was detected in only 27 isolates (18%). The majority of isolates (123 isolates, 82%) were non-hemolytic (γ-hemolysis) including all XDR isolates. Regarding motility testing, 102 isolates (68%) showed diffusion of growth with red color around the stab line representing motility. All XDR isolates were non-motile. Sixty-seven isolates (44.67%) were serum resistant, 4 isolates (2.67%) were intermediate serum resistant, and 21 isolates (14%) were slow-intermediate serum resistant, while 58 isolates (38.67%) were serum sensitive. Among XDR isolates, only one isolate was slow-intermediate serum resistant (Table S1). Statistical analysis has revealed no correlation between motility, hemolysin and serum resistance, and clinical source of the isolates (Table S2.A and Fig. S2).

#### Biofilm formation capacity

The capacity of biofilm formation among *E. coli* isolates was scored as strong, moderate, weak, and non-adherent isolates. Biofilm production was observed in most clinical isolates (134 isolates, 89.33%). The highest percent of the biofilm producing isolates was classified as weak biofilm producers 76 (50.6%), followed by moderate biofilm producers (51 isolates, 34%) and the least observed was strong producers (7 isolates, 4.67%) (Table S1).

**Fig. 3:**
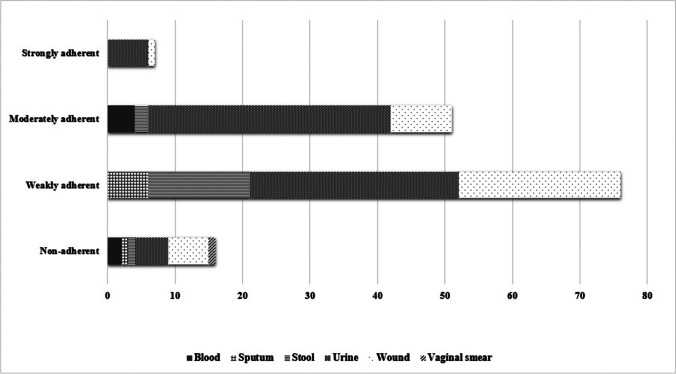
Distribution of biofilm forming capacities among *E. coli* isolates collected from different clinical sources

Statistical analysis has revealed correlation between biofilm formation capacity and the clinical source of the isolates (*P* < 0.05). Higher capacity of biofilm formation was recognized among *E. coli* isolated from urine, where 42 isolates (53.85%) were strong/moderate biofilm producers (Table S2.A, Fig. 3, and Fig. S2). However, most motile isolates had lower capacity of biofilm formation (*P* < 0.05). There was no significant correlation between hemolysin activity and serum resistance and the capacity of biofilm formation (Table S2.B and Fig. S2).

Among virulence determinants detected, isolates having P-fimbrial Usher Protein (*papC)*, Group 2 capsule system (*kpsMTII*), and Ferric yersiniabactin receptor (*fyuA*) had considerably greater biofilm formation capacity (Table S2.B). While, isolates harboring Shiga toxin II (*stx2)* and Cytotoxic necrotizing factor I *(cnf1*) showed weaker biofilm formation capacity. No significant correlation was observed between other genes detected and intensity of biofilm formation (Table S2.B and Fig. S3). To evaluate the association of different detected genes and biofilm formation capacity, we performed univariable logistic regression followed by multivariable logistic regression analysis (Table 2). Our results have showed association of strong/moderate biofilm formation capacity with P-fimbrial Usher Protein (*papC)*, Group 2 capsule system (*kpsMTII*), and Cytotoxic necrotizing factor I *(cnf1*) (*P* < 0.05, Table 2).

**Table 2:** Assessment of association between biofilm formation capacity and virulence determinants detected among *E. coli* clinical isolates

Biofilm formation	Variable	OR (95% CI)	*P*
A) Binary regression analysis			
Strong/moderate biofilm formation	***fyuA***	1.8191 (0.9231–3.5851)	0.0839
	***iutA***	1.263 (0.6357–2.5093)	0.5051
	***chuA***	1.5217 (0.7817–2.9625)	0.2167
	***Afa/dra***	0.5923 (0.2157–1.6262)	0.3095
	***sfaS***	0.9107 (0.4662–1.779)	0.7843
	***yfcV***	0.7413 (0.3652–1.5047)	0.4073
	***papC***	3.193 (1.6017–6.3654)	**0.001**
	**papG (allele II)**	1.1877 (0.5316–2.6534)	0.675
	***sta2***	1.9209 (0.7852–4.6993)	0.1526
	***stx2***	0.3765 (0.1915–0.7402)	**0.0046**
	***astA***	1.6687 (0.8552–3.2558)	0.1332
	***cnf1***	0.381 (0.188–0.7718)	**0.0074**
	***vat***	0.4337 (0.0869–2.1637)	0.3083
	***eae***	0.8914 (0.3488–2.2785)	0.8103
	***kpsMTII***	2.7468 (1.2609–5.9837)	**0.011**
B) Multiple regression analysis			
Strong/moderate biofilm formation	***papC***	2.8406 (1.3324–6.056)	**0.0069**
	***stx2***	0.5011 (0.2405–1.0443)	0.0651
	***cnf1***	0.3042 (0.1387–0.6671)	**0.003**
	***kpsMTII***	2.4799 (1.0468–5.875)	**0.039**

#### Resistance profiles of E. coli clinical isolates

High frequency of resistance was observed toward ampicillin (98%), ampicillin-sulbactam (97.33%), and amoxicillin-clavulanic acid (91.33%), while the lowest frequencies were observed against tigecycline (2%), fosfomycin (4.67%), and colistin-sulfate (7.33%) (Table S1). According to the resistance profile against tested antibiotics, 6 isolates (4%) were XDR and 139 isolates (92.67%) were MDR, while only 5 isolates (3.33%) were resistant to less than 3 classes of antibiotics and considered sensitive. No PDR isolates were detected among tested isolates (Table S1).

Statistical analysis has revealed significant correlation between biofilm formation capacity and susceptibility to different categories of antimicrobial agents including β-Lactam antibiotics, quinolones, aminoglycosides, colistin, and trimethoprim-sulphamethoxazole. Higher resistance rate was observed among strong/moderate biofilm producers (*P* < 0.05, Table 3). All XDR isolates showed strong/moderate adherence (Table S1). Furthermore, high diversity in resistance score (RS) was observed among all phylotypes. There was no significant association between phylotypes and RS (Table S3.A&C).

**Table 3:**
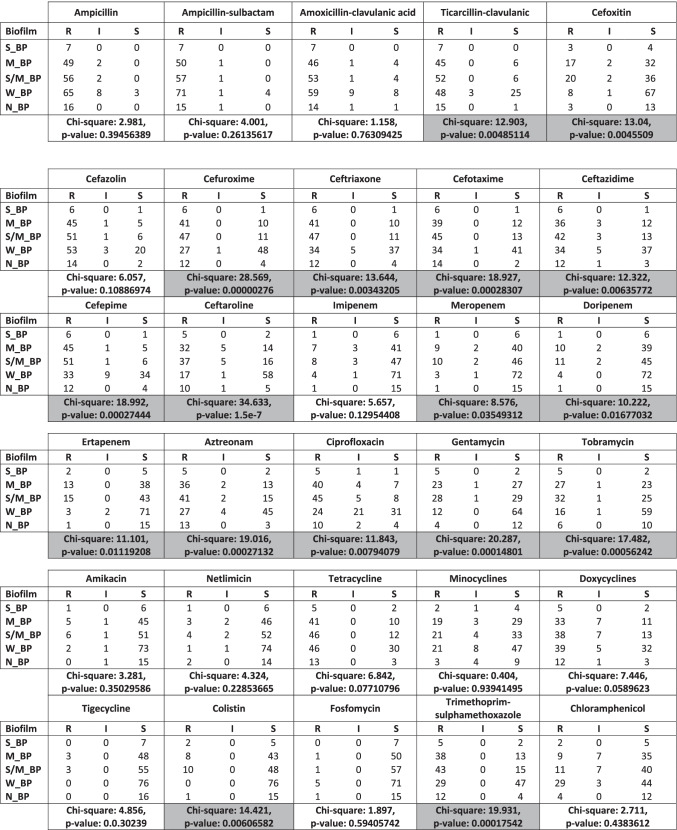
Correlation between biofilm formation capacity and resistance to different classes of antimicrobial agents among *E. coli* clinical isolates

## Discussion

*E. coli* seems to be an innocuous commensal normally inhabiting the gut of both humans and animals; however, a number of strains are pathogenic and were distinguished as a cause of food-borne enteritis (Blount 2015; Da Silva and Mendonça 2012). Numerous strains defy the threshold between commensal and pathogenic organisms; they intermittently cause extra-intestinal diseases in hosts as they inhabit their intestine asymptomatically primarily (Johnson and Russo 2002). They do not cause enteric manifestations but are serologically and phylogenically divergent from commensal and enteropathogenic strains. Therefore, *E. coli* can be categorized genetically and clinically into three major clusters: commensal, pathogenic, and extra-intestinal pathogenic *E. coli* (Kaper et al. 2004).

Bacteria can develop resistance to several classes of antibiotics through target site modification (mutation) and/or horizontal gene transfer. Mutation occurs spontaneously with variable frequency, based on the nature of the antibiotic and the microorganism, while horizontal gene transfer plays a key role in bacterial evolution and the spread of antimicrobial resistance determinants (Da Silva and Mendonça 2012). In our study, high rates of resistance to β-lactam, floroquinolones, aminoglycosides, and tetracyclines were detected among *E. coli* isolates collected from different clinical sources in Mansoura, Dakahlia governorate, Egypt. Moreover, resistance profiles of tested isolates have revealed dramatic increase of MDR (92.67%) and emergence of XDR (4%). Elevated rates of resistance to several classes of antimicrobial agents pose a serious health problem decreasing the treatment efficiency and limiting treatment options (Monroy-Pérez et al. 2020). Similar trends were recently reported in Egypt; where, Masoud et al. have reported that 73% of *E. coli* isolates collected from different clinical sources were MDR (Masoud et al. 2021). On the other hand, Khairy et al. have indicated MDR in 55% of *E. coli* isolated from children with community acquired-diarrhea in Upper Egypt (Khairy et al. 2020).

Elevated rates of antibiotic resistance among pathogenic *E. coli* strains worldwide have been also reported. However, the pattern of antimicrobials resistance varies by time and geographical region. Flament-Simon et al. have reported MDR in 37.2% of *E. coli*, causing urinary tract infections and other extraintestinal infections in Spain and France during 2016 (Flament-Simon et al. 2020). While, Duan et al. and Dehkordi et al. have reported similar rates of resistance to β-lactam, aminoglycosides, floroquinolones, and tetracyclines in China and Iran, respectively (Duan et al. 2020 and Dehkordi et al. 2020). Tigecycline, fosfomycin, and colistin remain the ultimate option for management of infections caused by XDR strains of *E. coli* (Abdelraouf et al. 2020; Malekzadegan et al. 2018; Vranic and Uzunovic 2016; Wang et al. 2018).

*E. coli* carries a wealth of virulence factors including biofilm formation, serum resistance, toxins production, iron acquisition systems, adhesins, and capsule that promote tissue colonization, damage, and foster distant diseases. These virulence traits allow the microorganism to overcome host defense mechanisms, colonize anatomical milieus, and provoke inflammatory response within the host initiating pathogenic diseases (Da Silva and Mendonça 2012). The expression of those virulence factors in *E. coli* plays a role in escalating their pathogenesis especially that most of them are MDR, rendering their treatment challenging (Ochoa et al. 2016).

Biofilm formation is an important virulence determinant that plays a major role to infections associated with numerous medical devices. The capability of microbes to form biofilm, where bacterial communities are embedded in exopolysaccharides, is one of the resistance mechanisms employed by several bacterial species to persist in the presence of antimicrobial agents. In the current study, the majority of *E. coli* clinical isolates were biofilm producers where 38.67% was strong/moderate biofilm producers. Moreover, higher prevalence of strong/moderate biofilm formation capacity was observed among isolates collected from urine (*P* < 0.05, Table S2 and Fig. S2). An earlier study has demonstrated that only 16% of MDR *E. coli* strains isolated from rectal swaps of post organ transplantation patients was strong/moderate biofilm producers (Ramos-Vivas et al. 2019). While, Cepas and coauthors have found that 30.3% of *E. coli* isolated from different sources has biofilm formation capacity, where isolates collected from respiratory source have less biofilm forming capacity than those from blood or urine (Cepas et al. 2019). A recent study in Egypt has denoted that 56.25% of *E. coli* isolates from diverse clinical sources was strong/moderate biofilm producers, where elevated rate of strong biofilm production was detected among *E. coli* recovered from urine (Abd El-Baky et al. 2020). Several studies that used microtiter plate for assessment of biofilm formation have shown varying capacities among UPEC strains (Abad et al. 2019; Karam et al. 2018; Noie Oskouie et al. 2019; Samet et al. 2013; Taghadosi et al. 2015; Zamani and Salehzadeh 2018). The collective rate of biofilm formation was 84.6%, where 50.9% was capable of producing strong/moderate biofilm and only 9.3% didn’t have the ability of biofilm production (Zhao et al. 2020).

Our results have revealed significant correlation between the intensity of biofilm formation and resistance to different categories of antimicrobial agents including β-Lactams, quinolones, aminoglycosides, colistin, and trimethoprim-sulphamethoxazole (Table 3). Moreover, all XDR showed strong/moderate adherence. The relation between the capacity of *E. coli* isolates to form biofilm and antimicrobial resistance was described previously. Cepas and coauthors have stated that resistance to ceftazidime and gentamicin was linked to biofilm formation capacity (Cepas et al. 2019), while Karam and coauthors have found correlation between ceftazidime, trimethoprim-sulphamethoxazole, norfloxacin, and biofilm formation capacity (Karam et al. 2018). Moreover, resistance to co-trimoxazole, tetracycline, norfloxacin, and ampcillin was comparatively superior among biofilm producers than biofilm non-producers (Ponnusamy et al. 2012).

The majority of *E. coli* isolates (82%) were non-hemolytic (γ-hemolysis) including all XDR isolates. Moreover, there was no substantial correlation between hemolysin activity and the source of isolates or the capacity of biofilm formation (Table S2). Morgan and coauthors have found that only 16.8% of UPEC isolates was hemolytic (Morgan et al. 2019). On the other hand, Abd El-Baky and coauthors have indicated that hemolytic activity was detected in 42.2% of *E. coli* isolates collected from different sources with significant higher rate among UPEC (Abd El-Baky et al. 2020). Also, Soto and coauthors have recorded a significantly higher frequency of hemolysin among biofilm forming UPEC isolates (Soto et al. 2007). Hemolytic activity may add to the virulence of *E. coli* through bloodstream infection and sepsis (Daga et al. 2019). The ability of *E. coli* strains to evade the complement system all through serum resistance accounts for the endurance of *E. coli* in the bloodstream; increased risk of developing septic shock and increased mortality (Micenková et al. 2017). In this study, 61.33% of *E. coli* isolates showed varying levels of serum resistance; however, there was no substantial correlation between serum resistance and the source of isolates or the intensity of biofilm formation (Table S2 and Fig. S2).

*E. coli* serotypes are specific O-group/H-antigen combinations, where H antigen is the flagellar one. Some *E. coli* isolates might be non-flagellated (non-motile) and so H negative (Naves et al. 2008). In the present study, 68% showed diffusion of growth with red color around the stab line representing motility. However, most motile isolates had lower capacity of biofilm formation (Table S2). Naves and coauthors have found no substantial correlation between motility and biofilm production (Naves et al. 2008). Nevertheless, such feature has formerly been considered crucial for biofilm formation (Van Houdt and Michiels 2005).

*E. coli* has an array of virulence determinants that contribute not only to its survival within the host but also to its pathogenesis as well. Iron is a vital element for all bacteria and catalyzes a broad range of vital enzymatic reactions; however, it is crucial to the host cells as well. Bacterial siderophore affinity to iron is superior to that of the host proteins; therefore, pathogens surpass the host in iron acquisition (Wilson et al. 2016). Siderophores are frequently linked to ExPEC particularly SEPEC (Bozcal et al. 2018; Koga et al. 2014). In the current study, Ferric yersiniabactin receptor (*fyuA*) was the most predominant (56.67%). It has been implicated in the adequate uptake of iron from bloodstream. Moreover, biofilm formation was substantially associated with the prevalence of *fyuA* gene (*p* < 0.05), but this finding was not observed for other iron genes. Similar results were described previously (Ananias and Yano 2008; Karam et al. 2018). Another study has indicated that Ferric aerobactin receptor (*iutA)* was significantly associated with strong biofilm production among UPEC strains (Kot et al. 2016).

In the present study, genes related to adhesins have been investigated including P fimbriae (*pap*C and *pap*G), Afimbriae/Dr- binding fimbriae (*afa/dra),* The major subunit of a putative fimbriae (*yfcV)*, and S fimbriae (*sfa*S). The expression of surface adhesins enhances the virulence of pathogenic *E. coli* by commencing close contact of the organism and the host cells. Distinctive microbial adhesins are adapted to inhabit certain niches (Sarowska et al. 2019). P fimbriae (Pyelonephritis-associated pili) are essential factor in mannose-resistant agglutination of human erythrocytes. Moreover, *PapG* (the fimbrial-tip adhesin) is held responsible for specificity of P fimbriae-mediated adherence and attaches to glycosphingolipids found in the renal epithelium. S fimbriae mediate binding of UPEC to human renal proximal-tubular cells and brain microvascular endothelial cells through binding to α-sialyl-2–3-β-galactoside. Therefore, S fimbriae are more commonly detected in *E. coli* recovered from patients with meningitis and neonatal sepsis (Malekzadegan et al. 2018; Rehman et al. 2017). In UPEC, receptors for P and S fimbriae are situated on the surface of epithelial cells of the urinary tract. While, S fimbrial adhesin is an important virulence factor in strains responsible for meningitis and sepsis (Sarowska et al. 2019). S fimbriae were the most prevalent adhesin among tested isolates, followed by P fimbriae. Similar results were documented previously (Daga et al. 2019).

Capsule (K antigen) is a key virulence factor of *E. coli* as it protects the cell from opsonophagocytosis and complement-mediated killing. The role that capsule plays in serum resistance in vitro and in animal models has been previously verified (Li et al. 2011; Mellata et al. 2003). *kpsMTII,* encoding group II capsule, was detected in 69.3% of the tested clinical isolates. Groups II capsule is the most prevalent among ExPEC including UTI, bloodstream, and meningitis isolates and infrequent among commensal *E. coli* (Monroy-Pérez et al. 2020; Ramírez-Castillo et al. 2018).

The heat-stable (ST) and heat-labile (LT) enterotoxins are crucial virulence factors in ETEC. Both toxins induce secretion of water and ions resulting in watery diarrhea. Epidemiological studies imply that strains producing ST and/or LT elicit the most severe diarrhea among children in developing countries (Wang et al. 2019). Two ST variants, *STIa* (*STaI* or *STp*) and *STIb* (*STaII* or *STh*), were found in human ETEC strains (Taxt et al. 2010). Enteroaggragative stable toxin (EAST1) was detected in 40.67% of the tested isolates. EAST1 has been infrequently associated with incidences of diarrhea in animals and humans (Dubreuil 2019). Moreover, variants of *astA* gene were detected in ExPEC from human and avian origins (Maluta et al. 2017). The cytotoxic necrotising factor 1 (CNF1) is a Rho GTPase protein toxin that promotes invasion into host cells. It is rarely detected in feces of children with diarrhea, but more common among ExPEC, including UTIs, bacteriaemia, and meningitis in neonates (Fabbri et al. 2010). In our study, *cnf1* gene was detected in 42% of *E. coli* clinical isolates. Varying rates of *cnf1* carriage in UPEC were indicated ranging from 7.8 to 29.6% (Dadi et al. 2020; Naves et al. 2008; Yılmaz and Aslantaş 2020); however, higher rates were reported among cervico-vaginal *E. coli* (CVEC) isolates (Monroy-Pérez et al. 2020). The vacuolating autotransporter toxin (*vat*) belongs to class II serine protease AT protein of *Enterobacteriaceae* (SPATE), which is cytotoxic to chicken embryonic fibroblasts and plays a role in avian cellulitis infection (Nichols et al. 2016). The *vat* gene was also detected in UPEC and plays a role in its virulence (Nichols et al. 2016; Ramírez-Castillo et al. 2018). However, vat gene was detected in only 6.67% of the tested *E. coli* clinical isolates.

Previous studies have denoted association between biofilm formation capacity of *E. coli* clinical isolates and expression of different virulence factors. Naves and coauthors have found that *hlyA*, *cnf1, papC* and *papG* alleles, *sfa/focDE*, and *focG* were more common among strong biofilm producers (Naves et al. 2008). Another research has showed that biofilm production was substantially correlated with the prevalence of *fyuA* and *hma* genes among UPEC (Karam et al. 2018). While, Kot and coauthors indicated that only aerobactin gene was highly considerably correlated with strong biofilm-producing UPEC strains (Kot et al. 2016). Wijetunge and coauthors have found that more than 70% NMEC strains tested carried *kpsMTII* gene, had the ability to invade human brain endothelial cells, and demonstrated high biofilm formation capacity (Wijetunge et al. 2015).

Various patterns of virulence factors and virulence encoding genes have been recognized among tested isolates (Table S1). ExPEC strains exhibit an array of virulence determinants that poster their ability of multiplication, attachment to host cells, internalization, adoption to different habitats within the host, and evasion of the host immune responses (Monroy-Pérez et al. 2020). It has been documented that P-fimbriae (*pap*C) contributes to urinary tract infections and subsequent bacteraemia (Subashchandrabose and Mobley 2015). Ananias and Yano have stated that the association of *fyu*A with *pap*C, attached to *kps*MTII or to another capsule or protectin, could be the minimal requirement for bacterial progression from a renal site of infection into the bloodstream of non-compromised patients (Ananias and Yano 2008). While, Surbeck and coauthors have indicated that *E. coli* isolates that harbor *vat*, *fyuA*, *chuA*, and *yfcV* virulence genes could competently colonize the urinary tract (Spurbeck et al. 2012).

Phylogenetic clustering of *E. coli* strains is important not only to picture *E. coli* populations but also to describe the correlation between phylotypes and virulence and resistance profiles and disease as well. In the current study, B2 group was the most prevalent among ExPEC follwed by B1 and E, while B1 was the most prevalent among intestinal ones. A and B1 phylotypes are the most commonly reported among commensal *E. coli* strains inhabiting human and animals GIT (Kaper 2005; Mosquito et al. 2015). Previous studies have indicated that B2 phylotype was the most prevalent among UPEC (Čurová et al. 2020; Dadi et al. 2020; Iranpour et al. 2015; Wijetunge et al. 2015; Yılmaz and Aslantaş 2020), while other have indicated that D phylotype is the most common one (Ramírez-Castillo et al. 2018; Sheikh et al. 2019). Moreover, B2, A, D, and C phylogroups were the most frequent among cervico-vaginal *E. coli* (CVEC) (Monroy-Pérez et al. 2020).

In this study, XDR isolates harbored only 4–8 virulence genes, showed strong/ moderate biofilm production, were non-motile and non-hemolytic, and most of them (5 out of 6) were serum sensitive. This is in agreement with the general notion that increased resistance in *E. coli* isolates can be coupled with decreased virulence as a result of survival fitness cost (Beceiro et al. 2013). Nonetheless, some MDR isolates exhibited high virulence scores, which proves that the opposite is true as well (Petty et al. 2014). Previous studies have indicated that antibiotic resistance is more frequent in pathogenic strains of *E. coli* compared to commensal ones (Petty et al. 2014). The connection between virulence and resistance could be attributed to the recurrent exposure of pathogenic strains to antibiotics and/or horizontal transfer of mobile genetic elements and plasmids and their role in dissemination of both virulence and resistance traits (da Cruz Campos et al. 2020). The existence of stains combining virulence and MDR among human clinical isolates is tormenting, limiting therapeutic choices, and raising public health concerns.

In summary, our research has revealed that virulence traits were distributed among distinctive phylotypes, which contributes to its pathogenesis in human. The elevated rates of antimicrobial resistance and emergence of MDR and XDR pose a serious health problem and limits available therapeutic options for management of infections caused by pathogenic *E. coli* isolates.

## Supplementary Information

Below is the link to the electronic supplementary material.

Supplementary file1 (PDF 1902 KB)

## Data Availability

All data generated and analyzed during this study are included in this published article and its supplementary information files.
