# Supplementary material for: Characterization of virulence determinants and phylogenetic background of multiple and extensively drug resistant Escherichia coli isolated from different clinical sources in Egypt
Source: Appl Microbiol Biotechnol. 2022 Jan 20;106(3):1279–98. doi: 10.1007/s00253-021-11740-x (PMC8816750; doi:10.1007/s00253-021-11740-x)
Supplement: Supplementary file 1 — Supplementary file1 (PDF 1902 KB) [file 253_2021_11740_MOESM1_ESM.pdf]

Applied Microbiology and Biotechnology

Characterization of virulence determinants and phylogenetic background of multiple and Extensively drug resistant *Escherichia coli* isolated from different clinical sources in Egypt.

Rana El-baz*, Heba Shehta Said*†, Eman Salama Abdelmegeed, Rasha Barwa†

Department of Microbiology and Immunology, Faculty of Pharmacy,

Mansoura University, Mansoura 35516, Egypt.

*** Both authors contributed equally to this work.**

† Corresponding author: Heba Shehta Said, Ph.D.

Department of Microbiology and Immunology, Faculty of Pharmacy, Mansoura University, Mansoura 35516, Egypt. E-mail: hebashehta@mans.edu.eg or hebashehta@yahoo.com.

And: Rasha Barwa, Ph.D.

Department of Microbiology and Immunology, Faculty of Pharmacy, Mansoura University, Mansoura 35516, Egypt. E-mail: rasha2000@gmail.com.

Table S1: Characterization of *E. coli* clinical isolates (clinical data, antibiotic resistance and virulence profiles).

Isolate No	Isolation source	Date of isolation	Phylotype	Phylotype Subgroup	Resistance profile	Resistance type	Resistance Score (RS)	Biofilm	Serum Resistance	Hemolysis (Qualitative)	Hemolysis (%)	Motility	Virulence genes Profile	Virulence genes Score
E1	Urine	10/10/17	C		AM, SAM, AMC, TIM, FOX, CZ, CXM, CTX, CAZ, CRO, FEP, ZOX, IPM, MEM, DOR, ETP, ATM, CIP, CN, TOB, TE, DO, CT, SXT	MDR	14	NP	R_S	γ	0	NM	*stx2, cnf1*	2
E2	Wound	10/10/17	A	A1	AM, SAM, AMC, TIM, CZ, CXM, CTX, CAZ, CRO, FEP, ZOX, ATM, CIP, CN, TOB, TE, MH, DO, FOS, C	MDR	12	WP	R_S	γ	0	M	*sfaS, astA, stx2, eae*	4
E3	Wound	10/10/17	A	A1	AM, SAM, AMC, TIM, CZ, CXM, CTX, CAZ, CRO, FEP, ZOX, ATM, CIP, CN, TOB, TE, MH, DO, FOS, SXT, C	MDR	13	NP	R_S	γ	0	M	*sfaS, stx2, eae*	3
E4	Wound	11/10/17	F		AM, SAM, AMC, TIM, CZ, CXM, CTX, CAZ, CRO, FEP, ZOX, ATM, CIP, TE, DO, SXT	MDR	10	NP	S	γ	0	M	*chuA, sfaS, yfcV, kpsMTII*	4
E5	Wound	11/10/17	D	D1	AM, SAM, AMC, TIM, CZ, CXM, CTX, CAZ, CRO, FEP, ZOX, ATM, TE, DO, SXT	MDR	9	NP	S	γ	0	M	*chuA, sfaS, stx2*	3
E6	Wound	11/10/17	F		AM, SAM, AMC, TIM, CZ, CXM, CTX, CAZ, CRO, FEP, ZOX, IPM, ATM, CIP, CN, TOB, TE, DO, SXT	MDR	12	WP	S	γ	0	M	*chuA, yfcV, sta2, stx2, cnf1, kpsMTII*	6
E7	Stool	14/10/2017	UN		AM, SAM, AMC, TIM, CTX, CAZ, ATM, TOB	MDR	6	NP	R_S	γ	0	M	*fyuA, Afa/dra, papC, eae, kpsMTII*	5
E8	Wound	14/10/2017	C		AM, SAM, AMC, TIM, CZ, CXM, CTX, CAZ, CRO, ATM, CIP, TE, MH, DO, SXT, C	MDR	10	NP	R_{SI}	γ	0	M	*sfaS, astA, cnf1*	3
E9	Sputum	14/10/2017	C		AM, SAM, AMC, TIM, CZ, CTX, CAZ, CRO, FEP, ATM, CIP, TE, MH, DO, SXT, C	MDR	10	WP	R_S	γ	0	NM	*fyuA, iutA, sfaS, stx2, cnf1, eae*	6
E10	Wound	14/10/2017	A	A0	AM, SAM, AMC, TIM, CZ, CIP, AK, TE, MH, DO, C	MDR	8	WP	R_{SI}	γ	0	M	*sfaS, sta2, stx2, cnf1, eae*	5
E11	Wound	16/10/2017	B2	$B2_3$	AM, SAM, AMC, TIM, FOX, CZ, CXM, CTX, CAZ, CRO, FEP, ZOX, ATM, CIP, TOB, TE, DO, SXT	MDR	12	NP	R_{SI}	β	72.73	M	*fyuA, chuA, sfaS, yfcV, papC, papG, cnf1, kpsMTII*	7
E12	Sputum	16/10/2017	C		AM, SAM, AMC, TIM, FOX, CZ, CXM, CTX, CAZ, CRO, FEP, ZOX, ATM, CIP, SXT	MDR	10	NP	R_S	γ	0	NM	*iutA, sfaS, stx2, cnf1, eae, kpsMTII*	6
E13	Blood	16/10/2017	B1		AM, SAM, AMC, TIM, CZ, CXM, CTX, CAZ, CRO, FEP, ZOX, ATM, CIP, TE, MH, DO, SXT	MDR	10	NP	S	γ	0	M	*sfaS, astA, sta2, stx2, cnf1, kpsMTII*	6
E14	Blood	16/10/2017	B2	$B2_2$	AM, SAM, AMC, TIM, CZ, CXM, CTX, CAZ, CRO, FEP, ZOX, ATM, CIP, TE, MH, DO, SXT	MDR	10	NP	S	γ	0	M	*fyuA, iutA, chuA, yfcV, papC, papG, astA, cnf1, kpsMTII*	8
E15	Wound	16/10/2017	C		AM, SAM, AMC, TIM, CZ, CAZ, CRO, ATM, CIP, TE, MH, DO, SXT, C	MDR	10	WP	R_S	γ	0	NM	*fyuA, papC, astA, stx2, cnf1*	5
E16	Wound	16/10/2017	C		AM, SAM, AMC, TIM, CZ, CXM, CTX, CAZ, CRO, FEP, ZOX, ATM, CIP, TE, MH, DO, SXT	MDR	10	NP	R_{SI}	γ	0	NM	*fyuA, sfaS, stx2*	3
E17	Urine	17/10/2017	E		AM, SAM, AMC, TIM, CZ, CXM, CTX, CAZ, CRO, FEP, ZOX, ATM, CIP, CN, TOB, NET, TE, DO, SXT	MDR	11	NP	S	γ	0	NM	*chuA, papC, stx2*	3

E18	Urine	17/10/2017	B1		AM, SAM, AMC, TIM, CZ, CTX, FEP, CIP, AK, TE, DO	MDR	8	NP	R_{SI}	8	0	M	*sta2, cnf1*	2
E19	Urine	20/10/2017	B2	$B2_3$	AM, SAM, AMC, TIM, TE, MH, DO, C	MDR	5	NP	S	β	62.12	NM	*fyuA, chuA, sfaS, yfcV, papC, papG, sta2, stx2, cnf1, kpsMTII*	9
E20	Vaginal smear	20/10/2017	A	A1	AM, SAM, CZ	MDR	3	NP	R_S	8	0	M	*sfaS, sta2, stx2, kpsMTII*	4
E21	Wound	20/10/2017	B2	$B2_3$	AM, SAM, AMC, TIM, FOX, CZ, CRO, FEP, TE, MH, DO, FOS, C	MDR	9	WP	R_S	β	78.79	M	*fyuA, chuA, sfaS, yfcV, papC, papG, stx2, cnf1, kpsMTII*	8
E22	Wound	20/10/2017	B2	$B2_3$	AM, SAM, AMC, TIM, FOX, CZ, TE, MH, DO, C	MDR	7	WP	R_S	β	54.55	NM	*fyuA, chuA, Afa/dra, sfaS, yfcV, papC, papG, stx2, cnf1, kpsMTII*	9
E23	Sputum	20/10/2017	B2	$B2_3$	AM, SAM, AMC, TIM, CZ, TE, MH, DO, C	MDR	6	WP	R_{SI}	β	7.58	NM	*fyuA, chuA, sfaS, yfcV, papC, papG, stx2, cnf1, kpsMTII*	8
E24	Urine	20/10/2017	B2	$B2_3$	AM, SAM, AMC, TIM, CZ, TE, MH, DO, C	MDR	6	WP	R_S	β	53.03	NM	*fyuA, chuA, Afa/dra, sfaS, yfcV, papC, papG, stx2, cnf1, vat, kpsMTII*	10
E25	Sputum	20/10/2017	B2	$B2_2$	AM, SAM, AMC, TIM, CZ, TE, MH, DO, C	MDR	6	WP	R_S	β	84.85	M	*fyuA, chuA, Afa/dra, yfcV, papC, papG, kpsMTII*	6
E26	Urine	20/10/2017	B2	$B2_3$	AM, SAM, AMC, TIM, FOX, CZ, CXM, CTX, CAZ, CRO, FEP, ZOX, IPM, ETP, ATM, CIP, CN, TE, MH, FOS, C	MDR	14	WP	R_S	β	39.39	NM	*fyuA, iutA, chuA, sfaS, yfcV, papC, papG, sta2, stx2, vat, kpsMTII*	10
E27	Urine	26/10/2017	B2	$B2_3$	AM, SAM, AMC, TIM, CRO, CIP	MDR	5	WP	R_S	8	0	M	*fyuA, iutA, chuA, Afa/dra, papC, sta2, eae, kpsMTII*	8
E28	Wound	26/10/2017	B2	$B2_3$	AMC, CZ, CTX, CAZ, CRO, FEP, ZOX, MEM, ETP, CIP, FOS, C	MDR	7	WP	R_{SI}	β	69.70	M	*fyuA, chuA, Afa/dra, sfaS, yfcV, papC, papG, kpsMTII*	7
E29	Wound	26/10/2017	B1		AM, SAM, AMC, TIM, FEP, MH, DO	MDR	5	WP	S	8	0	M	*sta2, stx2*	2
E30	Wound	26/10/2017	B1		AM, SAM, AMC, CZ, CXM, CTX, CAZ, CRO, FEP, DO	MDR	5	WP	R_{SI}	8	0	M	*astA, stx2, kpsMTII*	3
E31	Wound	26/10/2017	B2	$B2_2$	AM, SAM, AMC, TIM, CZ, CIP, TE, MH, DO, C	MDR	7	WP	R_I	β	92.42	M	*fyuA, chuA, sfaS, yfcV, papC, papG, stx2, cnf1, vat, kpsMTII*	9
E32	Wound	26/10/2017	B2	$B2_3$	AM, SAM, AMC, TIM, CZ, CTX, CAZ, CRO, FEP, ATM, TE, MH, DO, C	MDR	8	WP	R_S	8	0	M	*fyuA, iutA, chuA, yfcV, papC, papG, kpsMTII*	6
E33	Stool	28/10/2017	B1		AM, SAM, CIP, TE, SXT	MDR	5	WP	S	8	0	M	*iutA, Afa/dra, sfaS, papC, stx2, cnf1, kpsMTII*	7
E34	Wound	28/10/2017	B1		AM, SAM, AMC, TIM, CZ, CXM, CTX, CAZ, CRO, ATM, CIP, TE, MH, DO, C	MDR	9	WP	S	8	0	M	*fyuA, iutA, cnf1, kpsMTII*	4
E35	Wound	28/10/2017	B1		AM, SAM, CZ, CIP	MDR	4	WP	R_S	8	0	M	*sfaS, astA, stx2, cnf1, kpsMTII*	5
E36	Stool	02/11/17	D	D2	AM, SAM, CZ	MDR	3	WP	S	8	0	M	*chuA*	1

E37	Stool	02/11/17	B1		AM, SAM, AMC, FOX, CZ, CIP, TE, MH, DO, C	MDR	7	WP	S	8	0	NM	*sfaS, astA, stx2, eae*	4
E38	Stool	02/11/17	B1		AM, SAM, CZ, CIP	MDR	4	MP	R_{SI}	8	0	M	*sfaS, stx2*	2
E39	Stool	02/11/17	B1		AM, SAM, CZ, CIP, C	MDR	5	WP	R_S	8	0	M	*fyuA, sfaS, stx2, cnf1*	4
E40	Stool	02/11/17	B1		AM, SAM, AMC, CTX, CAZ, CRO, FEP, ZOX, ATM, CIP, TOB, C	MDR	8	WP	S	8	0	M	*stx2*	1
E41	Urine	02/11/17	A	A0	AM, SAM, AMC, TIM, TE, MH, C	MDR	5	WP	R_S	8	0	M	*sfaS, yfcV, astA, stx2, kpsMTII*	5
E42	Urine	02/11/17	C		AM, SAM, CIP	MDR	3	MP	R_S	8	0	NM	*stx2*	1
E43	Stool	02/11/17	B2	B2$_3$	AM, SAM, AMC, TIM, TE, MH, DO, C	MDR	5	WP	R_S	β	53.03	NM	*fyuA, chuA, sfaS, yfcV, papC, papG, stx2, cnf1, vat, kpsMTII*	9
E44	Urine	04/11/17	B1		AM, SAM	SDR	2	MP	R_S	8	0	M	*stx2*	1
E45	Urine	04/11/17	B1		AM, SAM, AMC, TIM, CZ, CXM, CTX, CAZ, CRO, FEP, ZOX, ATM, CIP, CN, TOB, NET, TE, MH, DO, SXT, C	MDR	12	NP	S	8	0	NM	*yfcV, stx2, eae, kpsMTII*	4
E46	Urine	04/11/17	B2	B2$_3$	AM, SAM, AMC, TIM, CTX, CAZ, CRO, FEP, CIP, TOB, TE	MDR	7	WP	R_{SI}	8	0	M	*fyuA, chuA, sfaS, yfcV, papC, papG, cnf1, eae, kpsMTII*	8
E47	Urine	04/11/17	A	A1	CN	SDR	1	WP	R_S	8	0	M	*fyuA, iutA, sfaS, yfcV, papC, stx2, cnf1, vat, kpsMTII*	9
E48	Urine	04/11/17	B2	B2$_3$	AM, SAM, CZ, CXM	MDR	3	WP	R_S	8	0	M	*fyuA, chuA, sfaS, yfcV, papC, papG, stx2, cnf1, vat, kpsMTII*	9
E49	Urine	07/11/17	B2	B2$_3$	AM, SAM, AMC, TIM, CZ, CXM, CTX, CAZ, CRO, FEP, CIP, TE, DO	MDR	7	WP	S	8	0	M	*fyuA, chuA, sfaS, yfcV, kpsMTII*	5
E50	Urine	07/11/17	B2	B2$_3$	AM, SAM, AMC, TIM, TE, DO	MDR	4	SP	S	β	66.67	M	*iutA, chuA, Afa/dra, sfaS, yfcV, papC, papG, astA, stx2, cnf1, vat, eae, kpsMTII*	12
E51	Urine	07/11/17	B2	B2$_3$	AM, SAM, AMC, TIM, FOX, CZ, CXM, CRO, FEP, CIP, C	MDR	8	MP	S	8	0	M	*fyuA, iutA, chuA, sfaS, astA, stx2, cnf1, eae, kpsMTII*	9
E52	Stool	09/11/17	A	A1	AM, SAM, AMC	SDR	2	WP	R_S	8	0	M	*fyuA, sfaS, yfcV, stx2, cnf1, kpsMTII*	6
E53	Wound	09/11/17	E		AM, SAM, AMC, TIM, CZ, CN, C	MDR	6	WP	S	8	0	M	*chuA, sfaS, papC, astA, stx2*	5
E54	Stool	09/11/17	C		AM, SAM, AMC, TIM, CZ, CXM, CTX, CRO, FEP, ATM, CIP, TE, DO, SXT, C	MDR	10	WP	R_S	8	0	M	*iutA, sfaS, yfcV, astA, stx2, kpsMTII*	6
E55	Urine	09/11/17	F		AM, SAM, CZ, TOB, SXT	MDR	5	WP	S	8	0	M	*iutA, chuA, Afa/dra, yfcV, astA, eae, kpsMTII*	7
E56	Wound	09/11/17	B2	B2$_2$	AM, SAM, AMC, TIM, CZ, CXM, CTX, CAZ, CRO, FEP, ZOX, ATM, CIP, CN, TOB, TE, MH, DO, SXT	MDR	11	MP	R_{SI}	8	0	M	*fyuA, iutA, chuA, sfaS, yfcV, astA, eae, kpsMTII*	8
E57	Stool	11/11/17	B2	B2$_3$	AM, SAM, AMC, CZ	MDR	3	WP	S	8	0	M	*chuA, astA, stx2*	3

E58	Stool	11/11/17	C		AM, SAM, AMC	SDR	2	WP	R_S	8	0	M	*sfaS*	1
E59	Stool	11/11/17	B1		AM, SAM, AMC, TIM, CZ, CXM, CTX, CAZ, CRO, FEP, ZOX, ETP, ATM, CIP, TE, MH, DO, SXT	MDR	11	WP	R_S	8	0	M	*fyuA, iutA, sfaS, papC, astA, cnf1, kpsMTII*	7
E60	Urine	16/11/2017	B2	$B2_2$	AM, SAM, AMC, TIM, FOX, CZ, CXM, CTX, CAZ, CRO, FEP, ZOX, IPM, MEM, DOR, ETP, ATM, CIP, CN, TOB, TE, MH, DO, SXT	MDR	13	MP	R_S	8	0	M	*chuA, sfaS, astA, sta2, stx2, cnf1*	6
E61	Wound	19/11/2017	C		AM, SAM, AMC, TIM, FOX, CZ, CXM, CTX, CAZ, CRO, FEP, ZOX, IPM, MEM, DOR, ATM, CIP, CN, TOB, TE, MH, DO, SXT	MDR	13	WP	R_S	8	0	NM	*fyuA, papC, astA, stx2, eae*	5
E62	Wound	19/11/2017	C		AM, SAM, AMC, TIM, CZ, CXM, CTX, CAZ, CRO, FEP, ZOX, ATM, CIP, CN, TOB, NET, SXT	MDR	10	SP	S	8	0	M	*fyuA, iutA, Afa/dra, yfcV, papC, cnf1, kpsMTII*	7
E63	Urine	19/11/2017	B2	$B2_3$	AM, SAM, AMC, CZ, CTX, CAZ, CRO, FEP, ATM, CIP	MDR	6	WP	S	8	0	M	*chuA, Afa/dra, papC, stx2*	4
E64	Wound	19/11/2017	B1		AM, SAM, AMC, TIM, CZ, CXM, CTX, CAZ, CRO, FEP, ZOX, ATM, CIP, TOB, AK, NET, TE, DO	MDR	10	WP	S	β	45.45	M	*fyuA, iutA, sfaS, yfcV, papC, astA, stx2, cnf1, kpsMTII*	9
E65	Wound	19/11/2017	B2	$B2_3$	AM, SAM, AMC, TIM, CZ, CXM, CTX, CAZ, CRO, FEP, ZOX, CIP, CN, TOB, TE, DO	MDR	9	MP	R_S	8	0	M	*iutA, chuA, astA, stx2, cnf1, kpsMTII*	6
E66	Wound	19/11/2017	B1		AM, SAM, AMC, TIM, CZ, CIP, TE, DO, SXT, C	MDR	8	MP	S	8	0	M	*iutA, astA*	2
E67	U.B drain	22/11/2017	A	A0	AM, SAM, AMC, TIM, CZ, CXM, CTX, CAZ, CRO, FEP, ZOX, ATM, TE, DO, SXT	MDR	9	MP	R_S	8	0	M	*iutA, Afa/dra, astA, kpsMTII*	4
E68	U.B drain	22/11/2017	E		AM, SAM, AMC, TIM, CZ, CXM, CTX, CAZ, CRO, FEP, CN, TOB, TE, DO, SXT	MDR	8	WP	R_S	β	5.30	NM	*iutA, chuA, yfcV, astA, stx2*	5
E69	Wound	23/11/2017	D	D1	AM, SAM, AMC, TIM, CZ, CXM, CTX, CAZ, CRO, FEP, ETP, ATM, CIP, CN, TOB, TE, DO	MDR	10	WP	S	β	1.52	M	*chuA, papC, astA*	3
E70	Wound	23/11/2017	A	A1	AM, SAM, AMC, TIM, FOX, CZ, CXM, CTX, CAZ, CRO, FEP, ZOX, IPM, MEM, DOR, ATM, CIP, TE, DO, SXT	MDR	12	WP	R_S	8	0	NM	*yfcV, cnf1, kpsMTII*	3
E71	Urine	23/11/2017	A	A1	AM, SAM, AMC, TIM, CZ, CXM, CTX, CAZ, CRO, FEP, ZOX, ATM, CIP, CN, TOB, TE, MH, DO, SXT	MDR	11	WP	R_S	8	0	NM	*iutA, sta2, stx2, cnf1*	4
E72	Wound	23/11/2017	C		AM, SAM, AMC, TIM, CZ, CXM, CTX, CAZ, CRO, FEP, ATM	MDR	6	WP	S	8	0	M	*iutA, sfaS, astA, cnf1*	4
E73	Stool	26/11/2017	B1		AM, SAM, AMC, CTX, CAZ, FEP, ATM	MDR	4	WP	R_S	β	3.03	M	*iutA, Afa/dra, papC, astA, stx2, cnf1*	6
E74	Stool	26/11/2017	B1		AM, SAM, AMC, TIM, CZ, DOR, SXT	MDR	6	WP	S	β	4.55	M	*fyuA, iutA, yfcV, papC, sta2, cnf1, kpsMTII*	7
E75	U.B drain	29/11/2017	A	A1	AM, SAM, AMC, CZ, CIP, C	MDR	5	WP	R_S I	8	0	M	*iutA, Afa/dra, stx2, kpsMTII*	4
E76	Stool	29/11/2017	B1		AM, SAM, AMC, CZ	MDR	3	WP	R_S	8	0	M	*Afa/dra, sfaS, papC, stx2*	4
E77	U.B drain	29/11/2017	B1		AM, SAM, AMC, TIM, FOX, CZ, CXM, CTX, CAZ, CRO, FEP, ZOX, ATM, CIP, TOB, TE, MH, DO, SXT, C	MDR	13	WP	S	8	0	M	*iutA, Afa/dra, sfaS, papC, astA*	5

E78	Urine	29/11/2017	B1		AM, SAM, AMC, TIM, FOX, CZ, CXM, CTX, CAZ, CRO, FEP, ZOX, IPM, MEM, DOR, ETP, ATM, CIP, CN, TOB, AK, NET, TE, MH, DO, SXT	MDR	13	WP	S	γ	0	M	*iutA, sfaS, papC, astA, stx2, cnf1, kpsMTII*	7
E79	U.B drain	29/11/2017	B1		AM, SAM, AMC, TIM, CZ, CXM, CTX, CAZ, CRO, FEP, ZOX, ATM, CIP, TE, MH, DO	MDR	9	WP	S	β	84.85	M	*fyuA, iutA, sfaS, yfcV, papC, papG, astA, cnf1*	7
E80	Stool	30/11/2017	B1		AM, SAM, AMC, CZ	MDR	3	WP	R_S	γ	0	M	*sfaS, astA, stx2, eae*	4
E81	U.B drain	02/12/17	B1		AM, SAM, AMC, CTX, CAZ, CRO, FEP, ATM	MDR	4	WP	R_S	γ	0	M	*sfaS, stx2, cnf1, vat, eae, kpsMTII*	6
E82	Wound	02/12/17	B1		AM, SAM, AMC, TIM, FOX, CZ, CXM, CTX, CAZ, CRO, FEP, ZOX, ATM, CIP, TOB, TE, DO, SXT	MDR	12	WP	R_S	γ	0	M	*fyuA, astA, cnf1, eae, kpsMTII*	5
E83	U.B drain	02/12/17	C		AM, SAM, AMC, TIM, CIP, TE, DO, SXT, C	MDR	7	WP	R_S	γ	0	M	*fyuA, iutA, sfaS, astA, cnf1, kpsMTII*	6
E84	swab throat	02/12/17	B2	$B2_2$	AM, SAM, AMC, TIM, CZ, CXM, CTX, CAZ, CRO, FEP, ZOX, ATM, CIP, TOB, TE, DO, SXT	MDR	11	WP	R_S	γ	0	M	*fyuA, iutA, chuA, astA, stx2, cnf1*	6
E85	Sputum	06/12/17	D	D1	AM, SAM, AMC, TIM, CZ, CXM, CTX, CAZ, CRO, FEP, ZOX, ATM, CIP, TOB, TE, DO, SXT	MDR	11	WP	R_S	β	1.52	M	*fyuA, iutA, chuA, yfcV, papC, papG, cnf1*	6
E86	Wound	06/12/17	B2	$B2_3$	AM, AMC, CZ, CTX, CAZ, CRO, FEP, ATM	MDR	4	WP	R_{SI}	β	12.12	M	*chuA, sfaS, astA, stx2, kpsMTII*	5
E87	Wound	06/12/17	B1		SAM, AMC, CIP	SDR	2	WP	S	γ	0	M	*Afa/dra, sfaS, stx2, kpsMTII*	4
E88	Wound	06/12/17	E		AM, SAM, AMC, TIM, CZ, CTX, CAZ, CRO, FEP, ATM, CIP, TE, DO, SXT, C	MDR	10	WP	R_S	γ	0	M	*fyuA, chuA, sfaS, astA, stx2, kpsMTII*	6
E89	Wound	06/12/17	F		AM, SAM, AMC, TIM, CZ, CXM, CTX, CAZ, CRO, FEP, ZOX, ATM, CIP, TE, DO	MDR	9	WP	R_{SI}	γ	0	NM	*fyuA, chuA, sfaS, stx2*	4
E90	Urine	10/12/17	B2	$B2_3$	AM, SAM, AMC, TIM, CZ, CXM, CTX, CAZ, CRO, FEP, ZOX, ATM, CIP, TE, DO	MDR	9	MP	R_{SI}	β	100	NM	*fyuA, chuA, sfaS, astA, stx2*	5
E91	U.B drain	10/12/17	B2	$B2_3$	AM, SAM, AMC, TIM, CAZ, FEP, ATM, CIP, TE, MH, DO, SXT, C	MDR	9	WP	S	γ	0	M	*fyuA, chuA, astA, stx2, kpsMTII*	5
E92	U.B drain	10/12/17	E		AM, SAM, AMC, TIM, CAZ, FEP, ATM, CIP, TE, MH, DO, SXT, C	MDR	9	WP	R_{SI}	γ	0	M	*fyuA, chuA, Afa/dra, sfaS, astA, stx2, cnf1*	7
E93	Urine	10/12/17	B2	$B2_3$	AM, SAM, AMC, TIM, CIP, TE, MH, DO, SXT, C	MDR	7	WP	R_S	γ	0	M	*fyuA, chuA, sfaS, astA, stx2, cnf1, kpsMTII*	7
E94	Urine	12/12/17	E		AM, SAM, AMC, TIM, CIP, TE, MH, DO, SXT, C	MDR	7	WP	R_S	γ	0	M	*fyuA, chuA, stx2, cnf1, kpsMTII*	5
E95	Urine	12/12/17	B1		AM, SAM, AMC, TIM, CAZ, FEP, CIP, TE, DO, SXT, C	MDR	8	WP	R_S	β	36.36	M	-	0
E96	Urine	12/12/17	E		AM, SAM, AMC, CZ	MDR	3	WP	R_{SI}	γ	0	M	*chuA, kpsMTII*	2
E97	Urine	12/12/17	B1		AM, SAM, AMC, TIM, CZ, CXM, CTX, CAZ, CRO, FEP, ZOX, ATM, CIP, CN, TOB, TE, DO, SXT	MDR	11	MP	R_S	γ	0	NM	*papC, papG, stx2*	2
E98	Urine	15/12/2017	F		AM, SAM, AMC, CZ, FEP	MDR	4	WP	R_S	γ	0	M	*fyuA, chuA, sfaS, yfcV, papC, stx2, kpsMTII*	7
E99	Urine	15/12/2017	D	D2	AM, SAM, AMC, TIM, CZ, CXM, CTX, CAZ, CRO, FEP, ZOX, ATM, CIP, TE, DO	MDR	9	WP	S	β	16.67	NM	*iutA, chuA, sfaS, stx2, kpsMTII*	5

E100	Urine	15/12/2017	F		AM, SAM, AMC, TIM, CZ, CXM, CTX, CAZ, CRO, FEP, ZOX, ATM, CIP, CN, TOB, SXT	MDR	10	MP	S	8	0	M	*fyuA, chuA, sfaS, yfcV, papC, papG, stx2, cnf1, kpsMTII*	8
E101	Urine	15/12/2017	B2	$B2_3$	AM, SAM, AMC, TIM, CZ, CXM, CTX, CAZ, CRO, FEP, SXT	MDR	6	WP	S	8	0	M	*fyuA, iutA, chuA, sfaS, stx2, cnf1, kpsMTII*	7
E102	Urine	27/4/2018	B2	$B2_3$	AM, AMC, TIM, CZ, TE, MH, DO, SXT	MDR	5	WP	S	8	0	M	*fyuA, iutA, chuA, sfaS, papC, papG, stx2, kpsMTII*	7
E103	Urine	27/4/2018	B2	$B2_3$	AM, SAM, AMC, TIM, CZ, CXM, CTX, CAZ, CRO, FEP, ZOX, ATM, CIP, TOB, TE, MH, DO, SXT	MDR	11	MP	R_S	8	0	M	*fyuA, iutA, chuA, yfcV, papC, papG, kpsMTII*	6
E104	Wound	28/4/2018	D	D1	AM, SAM, AMC, TIM, CZ, CXM, CTX, CAZ, CRO, FEP, ZOX, ATM, CIP, CN, TOB, TE, DO, SXT	MDR	11	MP	R_S	β	5.30	M	*fyuA, chuA, sfaS, yfcV, papC, papG, stx2, cnf1, kpsMTII*	8
E105	Urine	29/4/2018	E		AM, SAM, CZ, CXM, CTX, CAZ, CRO, FEP, CIP, SXT	MDR	6	WP	R_S	8	0	M	*fyuA, iutA, chuA, sfaS, stx2, cnf1, kpsMTII*	7
E106	Blood	03/05/18	Clade I		AM, SAM, AMC, TIM, FEP, ZOX, ATM, CN, TOB, TE, DO	MDR	8	MP	R_S	8	0	M	*fyuA, chuA, sfaS, yfcV, papC, papG, cnf1, kpsMTII*	7
E107	Urine	03/05/18	E		AM, SAM, AMC, TIM, CZ, CXM, CTX, CAZ, CRO, FEP, ZOX, ATM, CIP, TE, DO, SXT	MDR	10	MP	R_S	8	0	M	*fyuA, chuA, sfaS, stx2, kpsMTII*	5
E108	Throat swab	05/05/18	E		AM, SAM, AMC, TIM, CIP, CN, TOB, TE, MH, DO, SXT, C	MDR	8	WP	S	8	0	NM	*fyuA, chuA, sfaS, astA, cnf1, kpsMTII*	6
E109	Stool	05/05/18	E		AM, SAM, AMC, TIM, CZ, CXM, CTX, CAZ, CRO, ATM, CIP, TE, MH, DO, SXT, C	MDR	10	MP	S	8	0	M	*fyuA, chuA, sfaS, papC, cnf1, kpsMTII*	6
E110	Urine	09/05/18	B1		AM, SAM, AMC, FEP	MDR	3	MP	R_{SI}	8	0	M	*fyuA, Afa/dra, sfaS, papC, papG, stx2, cnf1, kpsMTII*	7
E111	Urine	09/05/18	B2	$B2_3$	AM, SAM, AMC, TIM, CZ, CXM, CAZ, CRO, FEP, ZOX, ATM, CIP, CN, TOB, TE, DO, FOS, SXT, C	MDR	13	MP	R_{SI}	8	0	M	*chuA, sfaS, yfcV, stx2, eae, kpsMTII*	6
E112	Urine	09/05/18	B2	$B2_3$	AM, SAM, AMC, TIM, CZ, FEP, CIP, TOB, TE, DO, SXT	MDR	9	MP	R_S	8	0	M	*chuA*	1
E113	Urine	09/05/18	Clade I		AM, SAM, AMC, TIM, CZ, CXM, CAZ, CRO, FEP, CIP, CN, TOB, TE, DO, FOS, SXT, C	MDR	11	WP	R_S	8	0	M	*fyuA, iutA, chuA, sfaS, yfcV, cnf1, kpsMTII*	7
E114	Urine	09/05/18	D	D2	AM, SAM, AMC, TIM, CZ, CXM, CRO, FEP	MDR	5	WP	S	8	0	M	*fyuA, chuA, yfcV, vat, kpsMTII*	5
E115	Urine	13/5/2018	E		AM, SAM, AMC, TIM, CZ, CXM, CAZ, CRO, FEP, ATM, CIP, CN, TOB, TE, DO, SXT	MDR	10	MP	S	β	77.27	M	*fyuA, iutA, chuA, sfaS, yfcV, papC, papG, kpsMTII*	7
E116	Urine	13/5/2018	Clade I		AM, SAM, AMC, TIM, CZ, CXM, CTX, CAZ, CRO, FEP, ZOX, ATM, CIP, TE, DO	MDR	9	MP	R_S	β	37.88	M	*chuA, sfaS, papC, papG, stx2, eae, kpsMTII*	6
E117	Urine	13/5/2018	B2	$B2_3$	AM, SAM, AMC, TIM, CZ, CXM, CAZ, CRO, FEP, ZOX, ATM, SXT	MDR	8	MP	R_S	β	54.55	NM	*fyuA, chuA, papC, kpsMTII*	4
E118	Urine	13/5/2018	D	D2	AM, SAM, AMC, CTX, FEP	MDR	3	MP	R_S	8	0	NM	*fyuA, chuA, kpsMTII*	3
E119	Urine	13/5/2018	E		AM, SAM, AMC, TIM, CZ, CXM, CTX, CAZ, CRO, FEP, CIP, SXT	MDR	7	SP	S	8	0	NM	*fyuA, iutA, chuA, sfaS, yfcV, papC, papG, kpsMTII*	7
E120	Urine	22/5/2018	C		AM, SAM, AMC, TIM, FOX, CZ, CXM, CTX, CAZ, CRO, FEP, ZOX, IPM, MEM, DOR, ETP, ATM, CIP, CN, TOB, TE, MH, DO, SXT	MDR	13	MP	R_S	8	0	NM	*fyuA, sfaS, papC, astA, kpsMTII*	5

E121	Urine	22/5/2018	B1		AM, SAM, AMC, TIM, CZ, CTX, FEP, CIP, MH, SXT, C	MDR	9	MP	R_S	8	0	NM	*iutA, papC*	2
E122	Urine	22/5/2018	F		AM, SAM, AMC, TIM, FOX, CZ, CXM, CTX, CAZ, CRO, FEP, ZOX, ETP, ATM, CIP, CN, TOB, TE, MH, DO, TGC, CT, SXT, C	XDR	16	SP	S	8	0	NM	*fyuA, chuA, sfaS, papC, astA, stx2, kpsMTII*	7
E123	Urine	22/5/2018	B1		AM, SAM, AMC, TIM, CZ, CXM, CTX, CAZ, CRO, FEP, ZOX, ATM, CIP, CN, TOB, TE, MH, DO	MDR	10	SP	S	8	0	NM	*fyuA, iutA, papC, stx2, kpsMTII*	5
E124	Urine	29/5/2018	B2	$B2_3$	AM, SAM, AMC, TIM, FOX, CZ, CXM, CTX, CAZ, CRO, FEP, ZOX, ATM, CIP, CN, TOB, AK, TE, MH, DO, SXT	MDR	12	SP	R_I	8	0	NM	*fyuA, chuA, astA, kpsMTII*	4
E125	Urine	29/5/2018	E		AM, SAM, AMC, TIM, FOX, CZ, CXM, CTX, CAZ, CRO, FEP, ZOX, IPM, MEM, DOR, ETP, ATM, CIP, CN, TOB, TE, DO, CT, SXT, C	XDR	15	SP	S	8	0	NM	*fyuA, chuA, sfaS, papC, astA, eae, kpsMTII*	7
E126	Urine	03/06/18	B1		AM, SAM, AMC, TIM, CZ, CXM, CTX, CAZ, CRO, FEP, ZOX, ATM, CIP, CN, TOB, TE, DO, SXT	MDR	11	MP	R_S	8	0	NM	*fyuA, iutA, yfcV, papC, kpsMTII*	5
E127	Urine	03/06/18	B1		AM, SAM, AMC, TIM, CZ, CXM, CTX, CAZ, CRO, FEP, ZOX, ATM, CIP, CN, TOB, TE, DO, SXT	MDR	11	MP	R_S	8	0	NM	*fyuA, sfaS, yfcV, papC, astA, stx2, kpsMTII*	7
E128	Urine	03/06/18	B1		AM, SAM, AMC, TIM, CZ, CXM, CTX, CAZ, CRO, FEP, ZOX, ATM, CIP, TOB, NET, TE, DO, SXT	MDR	11	MP	S	8	0	NM	*fyuA, iutA, sfaS, papC, papG, astA, sta2, stx2, cnf1, eae, kpsMTII*	10
E129	Urine	03/06/18	B1		AM, SAM, AMC, TIM, FOX, CZ, CXM, CTX, CAZ, CRO, FEP, ZOX, MEM, DOR, ETP, ATM, CIP, CN, TE, DO, CT, SXT	MDR	14	MP	S	8	0	M	*fyuA, iutA, sfaS, papC, astA, sta2, cnf1, kpsMTII*	8
E130	Urine	03/06/18	E		AM, SAM, AMC, TIM, FOX, CZ, CXM, CTX, CAZ, CRO, FEP, ZOX, ETP, ATM, CIP, CN, TOB, TE, MH, DO, SXT, C	MDR	14	MP	S	8	0	NM	*fyuA, chuA, sfaS, papC, astA, sta2*	6
E131	Urine	14/6/2018	B1		AM, SAM, AMC, TIM, CZ, CXM, CTX, CAZ, CRO, FEP, ZOX, ATM, CIP, TE, DO	MDR	9	MP	R_S	8	0	M	*fyuA, sfaS, astA, sta2, cnf1, kpsMTII*	6
E132	Urine	14/6/2018	B2	$B2_3$	AM, SAM, CZ, FEP	MDR	4	MP	R_S	8	0	NM	*fyuA, chuA, papC, astA, sta2, kpsMTII*	6
E133	Urine	14/6/2018	D	D1	AM, SAM, AMC, TIM, FOX, CZ, CXM, CTX, CAZ, CRO, FEP, ZOX, IPM, MEM, DOR, ETP, ATM, CIP, TE, MH, DO, CT, SXT, C	MDR	14	MP	S	8	0	M	*fyuA, iutA, chuA, sfaS, astA, sta2, stx2, kpsMTII*	8
E134	Urine	14/6/2018	E		AM, SAM, AMC, TIM, FOX, CZ, CXM, CTX, CAZ, CRO, FEP, ZOX, IPM, MEM, DOR, ETP, ATM, CIP, TE, MH, DO, SXT, C	MDR	13	MP	S	8	0	NM	*fyuA, chuA, papC, astA, sta2, stx2, kpsMTII*	7
E135	Urine	22/6/2018	D	D1	AM, SAM, AMC, TIM, FOX, CZ, CXM, CTX, CAZ, CRO, FEP, ZOX, IPM, MEM, DOR, ETP, ATM, CIP, TE, DO, C	MDR	12	MP	S	8	0	NM	*fyuA, chuA, yfcV, papC, astA, sta2, stx2, kpsMTII*	8
E136	Urine	22/6/2018	B1		AM, SAM, AMC, TIM, CZ, CXM, CTX, CRO, FEP, CIP, TE, MH, DO, SXT, C	MDR	9	MP	S	8	0	NM	*iutA, papC, astA, cnf1, kpsMTII*	5
E137	Urine	22/6/2018	B1		AM, SAM, AMC, TIM, CZ, CXM, CTX, CAZ, CRO, FEP, ZOX, ATM, CIP, TE, DO, SXT	MDR	10	MP	R_I	8	0	M	*fyuA, papC, papG, kpsMTII*	3
E138	Urine	22/6/2018	A	A1	AM, SAM, AMC, TIM, FOX, CZ, CXM, CTX, CAZ, CRO, FEP, ZOX, ATM, CIP, TOB, AK, NET, TE, DO, SXT	MDR	12	MP	S	8	0	M	*fyuA, papC, sta2, kpsMTII*	4

E139	Urine	22/6/2018	Clade I		AM, SAM, AMC, TIM, FOX, CZ, CXM, CTX, CAZ, CRO, FEP, ZOX, DOR, ETP, CIP, TOB, TE, MH, DO, SXT	MDR	12	MP	R_S	8	0	M	*fyuA, chuA, Afa/dra, sfaS, papC, kpsMTII*	6
E140	Urine	22/6/2018	A	A1	AM, SAM, AMC, TIM, FOX, CZ, CXM, CTX, CAZ, CRO, FEP, ZOX, ATM, CIP, CN, TOB, TE, MH, DO, CT, SXT, C	MDR	14	MP	S	8	0	NM	*sfaS, sta2, eae*	3
E141	Wound	28/6/2018	Clade I		AM, SAM, AMC, TIM, FOX, CZ, CXM, CTX, CAZ, CRO, FEP, ZOX, ATM, CIP, TOB, TE, MH, DO, SXT	MDR	12	MP	R_{SI}	8	0	M	*fyuA, chuA, papC, sta2, kpsMTII*	5
E142	Wound	28/6/2018	A	A1	AM, SAM, AMC, TIM, FOX, CZ, CXM, CTX, CAZ, CRO, FEP, ZOX, IPM, MEM, DOR, ETP, ATM, CIP, CN, TOB, AK, NET, TE, MH, DO, TGC, SXT, C	XDR	15	MP	S	8	0	NM	*fyuA, iutA, astA, kpsMTII*	4
E143	Blood	28/6/2018	A	A1	AM, SAM, AMC, TIM, CZ, CXM, CTX, CAZ, CRO, FEP, ZOX, ATM, CIP, CN, TOB, AK, TE, MH, DO, SXT	MDR	11	MP	S	8	0	M	*fyuA, iutA, sfaS, yfcV, papC, astA, kpsMTII*	7
E144	Urine	01/07/18	E		AM, SAM, AMC, TIM, FOX, CZ, CXM, CTX, CAZ, CRO, FEP, ZOX, IPM, MEM, DOR, ETP, ATM, CIP, CN, TOB, TE, MH, DO, SXT	MDR	13	MP	S	8	0	M	*iutA, chuA, papC, stx2, kpsMTII*	5
E145	Wound	17/7/2018	B2	$B2_3$	AM, SAM, AMC, TIM, CZ, CXM, CTX, CAZ, CRO, FEP, ATM, CIP, CN, TE, MH, DO, CT, SXT, C	MDR	12	MP	R_S	8	0	M	*iutA, chuA, sfaS, yfcV, papC, astA, cnf1, kpsMTII*	8
E146	Wound	17/7/2018	B2	$B2_3$	AM, SAM, AMC, TIM, FOX, CZ, CXM, CTX, CAZ, CRO, FEP, ZOX, IPM, MEM, DOR, ETP, ATM, CIP, CN, TOB, AK, NET, TE, MH, CT, SXT, C	XDR	15	MP	S	8	0	NM	*chuA, sfaS, yfcV, papC, papG, kpsMTII*	5
E147	Blood	17/7/2018	D	D2	AM, SAM, AMC, TIM, FOX, CZ, CXM, CTX, CAZ, CRO, FEP, ZOX, IPM, MEM, DOR, ETP, ATM, CIP, CN, TOB, AK, NET, TE, MH, DO, SXT	MDR	13	MP	R_S	8	0	NM	*fyuA, chuA, sfaS, yfcV, papC, astA, kpsMTII*	7
E148	Wound	17/7/2018	C		AM, SAM, AMC, TIM, FOX, CZ, CXM, CTX, CAZ, CRO, FEP, ZOX, IPM, MEM, DOR, ETP, ATM, CIP, CN, TOB, AK, TE, MH, DO, CT, SXT, C	XDR	15	MP	S	8	0	NM	*Afa/dra, sfaS, papC, vat, kpsMTII*	5
E149	Urine	03/08/18	B2	$B2_3$	AM, SAM, AMC, TIM, FOX, CZ, CXM, CTX, CAZ, CRO, FEP, ZOX, ATM, CIP, CN, TOB, TE, MH, DO, TGC, CT, SXT, C	XDR	15	MP	R_{SI}	8	0	NM	*iutA, chuA, sfaS, papC, astA, kpsMTII*	6
E150	Blood	06/08/18	D	D2	AM, SAM, AMC, TIM, FOX, CZ, CXM, CTX, CAZ, CRO, FEP, ZOX, ATM, CIP, CN, TOB, TE, MH, DO, CT, SXT	MDR	13	MP	R_I	8	0	NM	*fyuA, chuA, sfaS, papC, astA, stx2, cnf1, kpsMTII*	8

CN: Gentamicin, TOB: Tobramycin, AK: Amikacin, NET: Netilmicin, ZOX: Ceftaroline, TIM: Ticarcillin-clavulanate, ETP: Ertapenem, IPM: Imipenem, MEM: Meropenem, DOR: Doripenem, CZ: Cefazolin, CXM: Cefuroxime, CTX: Cefotaxime, CRO: Ceftriaxone, CAZ: Ceftazidime, FEP: Cefepime, FOX: Cefoxatin, CIP: Ciprofloxacine, SXT: Trimethoprim-sulphamethoxazole, TGC: Tigecycline, ATM: Aztreonam, AM: Ampicillin, AMC: Amoxicillin-clavulanic acid, SAM: Ampicillin-sulbactam, C: Chloramphenicol, FOS: Fosfomycin, CT: Colistin-sulphate, TE: Tetracycline, DO: Doxycycline, MH: Minocycline, MDR: Multidrug resistance, XDR: Extreme drug resistance, SDR: Sensitive. Resistance score (RS) is the number of antimicrobial classes to which resistance was detected. Biofilm: NP: Non-Biofilm Producer, WP: Weak Biofilm Producer, MP: Moderate Biofilm Producer, SP: Strong Biofilm Producer. Serum Resistance: R_S: Serum Resistance, R_I: Intermediate Serum Resistance, R_{SI}: Slow-Intermediate Serum Resistance. Motility: NM: Non-motile, M: Motile. Virulence gene score is the number of virulence genes/operons detected (adjusted for multiple detection of *pap, sfa* or *foc*, and *kpsM II* operons).

Table S2: Correlation between clinical source of *E. coli* isolates, phylotypes, virulence factors and genes detected in this study.

A. Correlation between clinical source of *E. coli* isolates and phylotypes, virulence factors and genes detected.

	Clermont Phylotypes								Clermont Phylotypes/subgroups									
**Source**	**A**	**B1**	**B2**	**C**	**Clade I**	**D**	**E**	**F**	**A0**	**A1**	**B1**	**$B2_2$**	**$B2_3$**	**C**	**D1**	**D2**	**E**	**F**
**Blood**	1	1	2	0	1	1	0	0	0	1	1	1	1	0	0	1	0	0
**Sputum**	0	0	3	2	0	1	1	0	0	0	0	2	1	2	1	0	1	0
**Stool**	1	10	2	2	0	1	1	0	0	1	10	0	2	2	0	1	1	0
**Urine**	7	19	23	4	3	5	13	4	2	5	19	1	22	4	2	3	13	4
**Vaginal smear**	1	0	0	0	0	0	0	0	0	1	0	0	0	0	0	0	0	0
**Wound**	5	8	10	7	1	4	2	3	1	4	8	2	8	7	3	1	2	3
All	Chi-square: 42.166, p-value: 0.18870377								**Chi-square: 61.962, p-value: 0.04736**									
All except vaginal smear	Chi-square: 33.058, p-value: 0.23361528								Chi-square: 50.701, p-value: 0.05294									

	Biofilm				Motility		Hemolysin		Serum Resistance		Serum Resistance			
**Source**	**S_BP**	**M_BP**	**W_BP**	**N_BP**	**M**	**NM**	**+**	**-**	**R**	**S**	**R_S**	**R_I**	**R_{SI}**	**S**
**Blood**	0	4	0	2	4	2	0	6	2	4	1	1	0	4
**Sputum**	0	0	6	1	3	4	3	4	6	1	5	0	1	1
**Stool**	0	2	15	1	16	2	3	15	11	7	10	0	1	7
**Urine**	6	36	31	5	47	31	12	66	46	32	35	2	9	32
**Vaginal Smear**	0	0	0	1	1	0	0	1	1	0	1	0	0	0
**Wound**	1	9	24	6	31	9	9	31	26	14	15	1	10	14
All	**Chi-square: 40.234, p-value: 0.000418**				Chi-square: 9.927, p-value: 0.07733		Chi-square: 5.399, p-value: 0.369148		Chi-square: 4.779, p-value: 0.44344239		Chi-square: 17.533, p-value: 0.288012			
All except vaginal smear	**Chi-square: 31.948, p-value: 0.00140958**				Chi-square: 9.42, p-value: 0.05142		Chi-square: 0.369, p-value: 0.6877		Chi-square: 4.134, p-value: 0.38817457		Chi-square: 16.224, p-value: 0.18119			

	fyuA		***iutA***		***chuA***		***afa/dra***		***sfaS***		***yfcV***	
**Source**	**+**	**-**	**+**	**-**	**+**	**-**	**+**	**-**	**+**	**-**	**+**	**-**
**Blood**	4	2	2	4	4	2	0	6	5	1	4	2
**Sputum**	6	1	4	3	5	2	1	6	4	3	3	4
**Stool**	7	11	5	13	4	14	4	14	12	6	4	14
**Urine**	48	30	31	47	48	30	11	67	44	34	24	54
**Vaginal smear**	0	1	0	1	0	1	0	1	1	0	0	1
**Wound**	20	20	10	30	20	20	5	35	25	15	15	25
All	Chi-square: 7.752, p-value: 0.17044		Chi-square: 5.012, p-value: 0.414417		**Chi-square: 11.777, p-value: 0.03797**		Chi-square: 2.226, p-value: 0.81707		Chi-square: 2.897, p-value: 0.71586		Chi-square: 5.329, p-value: 0.37706	
All except vaginal smear	Chi-square: 6.449, p-value: 0.16803		Chi-square: 4.464, p-value: 0.346837		**Chi-square: 10.608, p-value: 0.0313414**		Chi-square: 2.051, p-value: 0.72638		Chi-square: 2.239, p-value: 0.691896		Chi-square: 4.81, p-value: 0.30735	

	papC		***papG (allele II)***		***sta2***		***stx2***		***cnf1***		***vat***	
**Source**	**+**	**-**	**+**	**-**	**+**	**-**	**+**	**-**	**+**	**-**	**+**	**-**
**Blood**	5	1	3	3	1	5	2	4	4	2	0	6
**Sputum**	3	4	3	4	0	7	4	3	6	1	0	7
**Stool**	8	10	1	17	1	17	12	6	8	10	1	17
**Urine**	42	36	17	61	16	62	44	34	27	51	7	71
**Vaginal smear**	0	1	0	1	1	0	1	0	0	1	0	1
**Wound**	17	23	7	33	4	36	22	18	18	22	2	38
All	Chi-square: 5.393, p-value: 0.369822		Chi-square: 8.324, p-value: 0.13926		Chi-square: 10.611, p-value: 0.0596621		Chi-square: 2.876, p-value: 0.719096		Chi-square: 9.652, p-value: 0.0857		Chi-square: 1.882, p-value: 0.86522	
All except vaginal smear	Chi-square: 4.387, p-value: 0.356157		Chi-square: 8.022, p-value: 0.090776		Chi-square: 5.212, p-value: 0.266228		Chi-square: 2.103, p-value: 0.7168		Chi-square: 8.907, p-value: 0.063466		Chi-square: 1.799, p-value: 0.77267	

	astA		*eae*		*kpsMTII*	
**Source**	**+**	**-**	**+**	**-**	**+**	**-**
**Blood**	4	2	0	6	6	0
**Sputum**	2	5	2	5	4	3
**Stool**	6	12	3	15	8	10
**Urine**	30	48	12	66	59	19
**Vaginal Smear**	0	1	0	1	1	0
**Wound**	19	21	6	34	26	14
All	Chi-square: 4.123, p-value: 0.53184643		Chi-square: 2.241, p-value: 0.81489		**Chi-square: 10.642, p-value: 0.05896**	
All except vaginal smear	Chi-square: 3.426, p-value: 0.48921903		Chi-square: 2.047, p-value: 0.72711		**Chi-square: 10.159, p-value: 0.037833**	

B. Correlation between biofilm formation capacity and virulence factors and virulence genes detected.

	Motility		Hemolysin		Serum Resistance		Serum Resistance			
**Biofilm**	**M**	**NM**	**+**	**-**	**R**	**S**	**R_S**	**R_I**	**R_{SI}**	**S**
**S_BP**	2	5	1	6	1	6	0	1	0	6
**M_BP**	29	22	5	46	32	19	23	2	7	19
**S/M_BP**	31	27	6	52	33	25	23	3	7	25
**W_BP**	61	15	19	57	50	26	39	1	10	26
**N_BP**	10	6	2	14	9	7	5	0	4	7
	**Chi-square: 11.119, p-value: 0.0038507**		Chi-square: 5.154, p-value: 0.07600167		Chi-square: 1.292, p-value: 0.52413814		Chi-square: 6.404, p-value: 0.37948649			

	fyuA		*iutA*		*chuA*		*afa/dra*		*sfaS*		*yfcV*		*papC*	
**Biofilm**	**+**	**-**	**+**	**-**	**+**	**-**	**+**	**-**	**+**	**-**	**+**	**-**	**+**	**-**
**S_BP**	6	1	4	3	5	2	2	5	4	3	3	4	6	1
**M_BP**	32	19	18	33	30	21	4	47	30	21	14	37	33	18
**S/M_BP**	38	20	22	36	35	23	6	52	34	24	17	41	39	19
**W_BP**	42	34	28	48	40	36	14	62	47	29	28	48	31	45
**N_BP**	5	11	2	14	6	10	1	15	10	6	5	11	5	11
	**Chi-square: 6.12, p-value: 0.046888**		Chi-square: 3.903, p-value: 0.142061		Chi-square: 2.751, p-value: 0.252713		Chi-square: 2.676, p-value: 0.26237		Chi-square: 0.168, p-value: 0.919431		Chi-square: 0.875, p-value: 0.645649		**Chi-square: 11.725, p-value: 0.002844**	

	papG (allele II)		*sta2*		*stx2*		*cnf1*		*vat*		*eae*		*astA*	
**Biofilm**	**+**	**-**	**+**	**-**	**+**	**-**	**+**	**-**	**+**	**-**	**+**	**-**	**+**	**-**
**S_BP**	2	5	0	7	3	4	4	4	1	6	2	5	7	0
**M_BP**	11	40	12	39	21	30	24	24	1	50	6	45	40	11
**S/M_BP**	13	45	12	46	24	34	28	28	2	56	8	50	47	11
**W_BP**	15	61	7	69	51	25	30	30	8	68	11	65	48	28
**N_BP**	3	13	4	12	10	6	3	3	0	16	4	12	9	7
	Chi-square: 0.184, p-value: 0.9121		Chi-square: 4.628, p-value: 0.09887		**Chi-square: 9.11, p-value: 0.01049**		**Chi-square: 8.08, p-value: 0.01764**		Chi-square: 3.928, p-value: 0.140296		Chi-square: 1.301, p-value: 0.521785		Chi-square: 4.622, p-value: 0.09916	

	kpsMTII	
**Biofilm**	**+**	**-**
**S_BP**	7	0
**M_BP**	40	11
**S/M_BP**	47	11
**W_BP**	48	28
**N_BP**	9	7
	**Chi-square: 6.386, p-value: 0.041049**	

Table S3: Distribution of virulence score (VS) and resistance score (RS) and different phylotypes among *E. coli* clinical isolates.

A. Distribution of VS and RS among different phylotypes of *E. coli* clinical isolates.

	A	B1	B2	C	D	E	F	Clade I	Kruskal Wallis test
**VS**	4 (3-9)	5 (0-10)	7 (1-12)	5 (1-7)	5.5 (1-8)	6 (2-7)	7 (4-8)	6 (5-7)	**The test statistic *H* is 27.4052, The p-value is 0.0002817**
**RS**	11 (1-15)	9 (2-12)	7.5 (4-14)	10 (2-15)	10.5 (3-14)	10 (4-15)	10 (5-16)	11 (8-12)	The test statistic *H* is 6.7024, The p-value is 0.4605

VS and RS are presented as Median (Range).

B. Virulence score (VS) in relation to different phylotypes of *E. coli* clinical isolates.

	B1	B2	C	D	E	F	Clade I
**A**	The H statistic is 0.1229, The p-value is 0.7259	**The H statistic is 12.603, The p-value is 0.0003851**	The H statistic is 0.03613, The p-value is 0.8492	The H statistic is 0.844, The p-value is 0.3583	The H statistic is 5.327, The p-value is 0.021	The H statistic is 4.0703, The p-value is 0.04364	The H statistic is 5.2378, The p-value is 0.0221
**B1**		**The H statistic is 14.084, The p-value is 0.0001748**	The H statistic is 0.1754, The p-value is 0.6753	The H statistic is 1.0851, The p-value is 0.2976	The H statistic is 2.827, The p-value is 0.09269	The H statistic is 2.7196, The p-value is 0.09912	The H statistic is 2.4696, The p-value is 0.1161
**B2**			**The H statistic is 12.1047, The p-value is 0.000503**	The H statistic is 2.6425, The p-value is 0.104	The H statistic is 3.9946, The p-value is 0.04565	The H statistic is 0.6422, The p-value is 0.4229	The H statistic is 0.5633, The p-value is 0.453
**C**				The H statistic is 1.5919, The p-value is 0.207	The H statistic is 4.3714, The p-value is 0.03655	The H statistic is 4.2903, The p-value is 0.03833	The H statistic is 4.4099, The p-value is 0.03573
**D**					The H statistic is 0.01273, The p-value is 0.9102	The H statistic is 0.1846, The p-value is 0.6675	The H statistic is 0.1033, The p-value is 0.7479
**E**						The H statistic is 0.6777, The p-value is 0.4104	The H statistic is 0.4303, The p-value is 0.5119
**F**							The H statistic is 0.06502, The p-value is 0.7987

Kruskal Wallis test was used to compare the VS among different phylotypes followed by Mann Whitney U test for each pair of groups.
The p-value was adjusted for multiple comparisons using Bonferroni correction method. (The corrected α using Bonferroni correction method is 0.00625. The significant p-values after correction are in red).

C. Resistance score (RS) in relation to different phylotypes of *E. coli* clinical isolates.

	B1	B2	C	D	E	F	Clade I
**A**	The H statistic is 1.0038, The p-value is 0.3164	The H statistic is 0.5623, The p-value is 0.4533	The H statistic is 0.01092, The p-value is 0.9168	The H statistic is 0.02163, The p-value is 0.8831	The H statistic is 0.001435, The p-value is 0.9698	The H statistic is 0.01128, The p-value is 0.9154	The H statistic is 0.09519, The p-value is 0.7577
**B1**		The H statistic is 0.00002524, The p-value is 0.996	The H statistic is 2.1358, The p-value is 0.1439	The H statistic is 1.726, The p-value is 0.1889	The H statistic is 1.6184, The p-value is 0.2033	The H statistic is 0.9852, The p-value is 0.3209	The H statistic is 2.3845, The p-value is 0.1225
**B2**			The H statistic is 2.0561, The p-value is 0.1516	The H statistic is 1.2393, The p-value is 0.2656	The H statistic is 2.1446, The p-value is 0.1431	The H statistic is 0.9834, The p-value is 0.3214	The H statistic is 2.6143, The p-value is 0.1059
**C**				The H statistic is 0.005522, The p-value is 0.9408	The H statistic is 0.08397, The p-value is 0.772	The H statistic is 0.04803, The p-value is 0.8265	The H statistic is 0.09759, The p-value is 0.7547
**D**					The H statistic is 0.07138, The p-value is 0.7893	The H statistic is 0.007219, The p-value is 0.9323	The H statistic is 0.01129, The p-value is 0.9154
**E**						The H statistic is 0.001028, The p-value is 0.9744	The H statistic is 0.5609, The p-value is 0.4539
**F**							The H statistic is 0.2424, The p-value is 0.6224

Kruskal Wallis test was used to compare the RS among different phylotypes followed by Mann Whitney U test for each pair of groups.
The p-value was adjusted for multiple comparisons using Bonferroni correction method. (The corrected α using Bonferroni correction method is 0.00625).

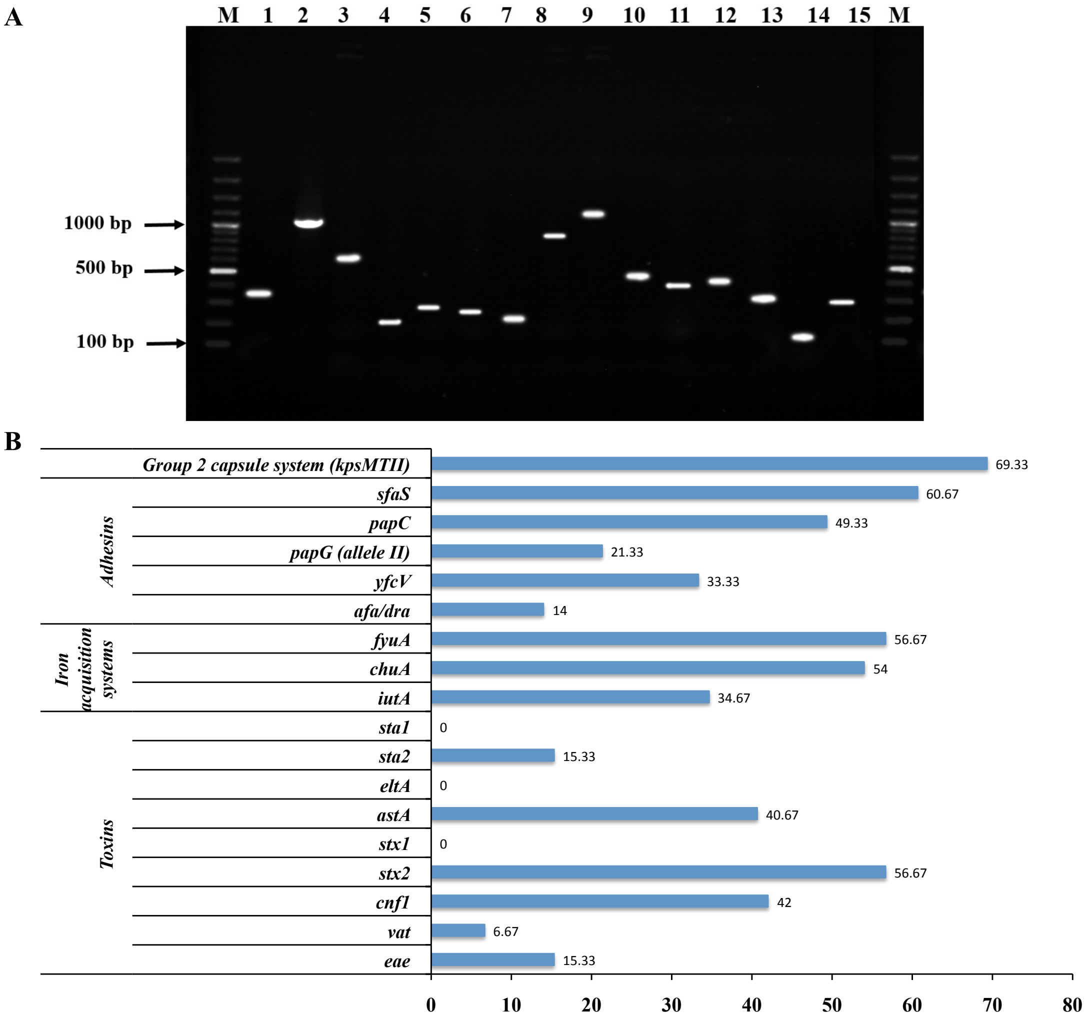

Fig. S1: Detection of virulence encoding genes among *E. coli* clinical isolates.

A. Agarose gel electrophoresis for detection of virulence encoding genes among *E. coli* clinical isolates by PCR. Lane M is Molecular weight marker. *1: papC* (350 bp); 2: *papG allele II* (1070 bp); 3: *afa/dra* (592 bp); 4: *sfaS* (240 bp); 5: *yfcV* (292 bp); *6: kpsMTII* (272 bp)*;* 7: *iutA* (253 bp); 8: *fyuA* (880 bp); 9: *vat* (1100 bp); 10: *cnf1 (498 bp); 11: stx2 (380 bp); 12: eae (384 bp); 13: sta2 (300 bp); 14: astA (106 bp); and 15: chuA (288 bp)* genes.

B. Bar chart representing prevalence of virulence encoding genes among *E. coli* clinical isolates.

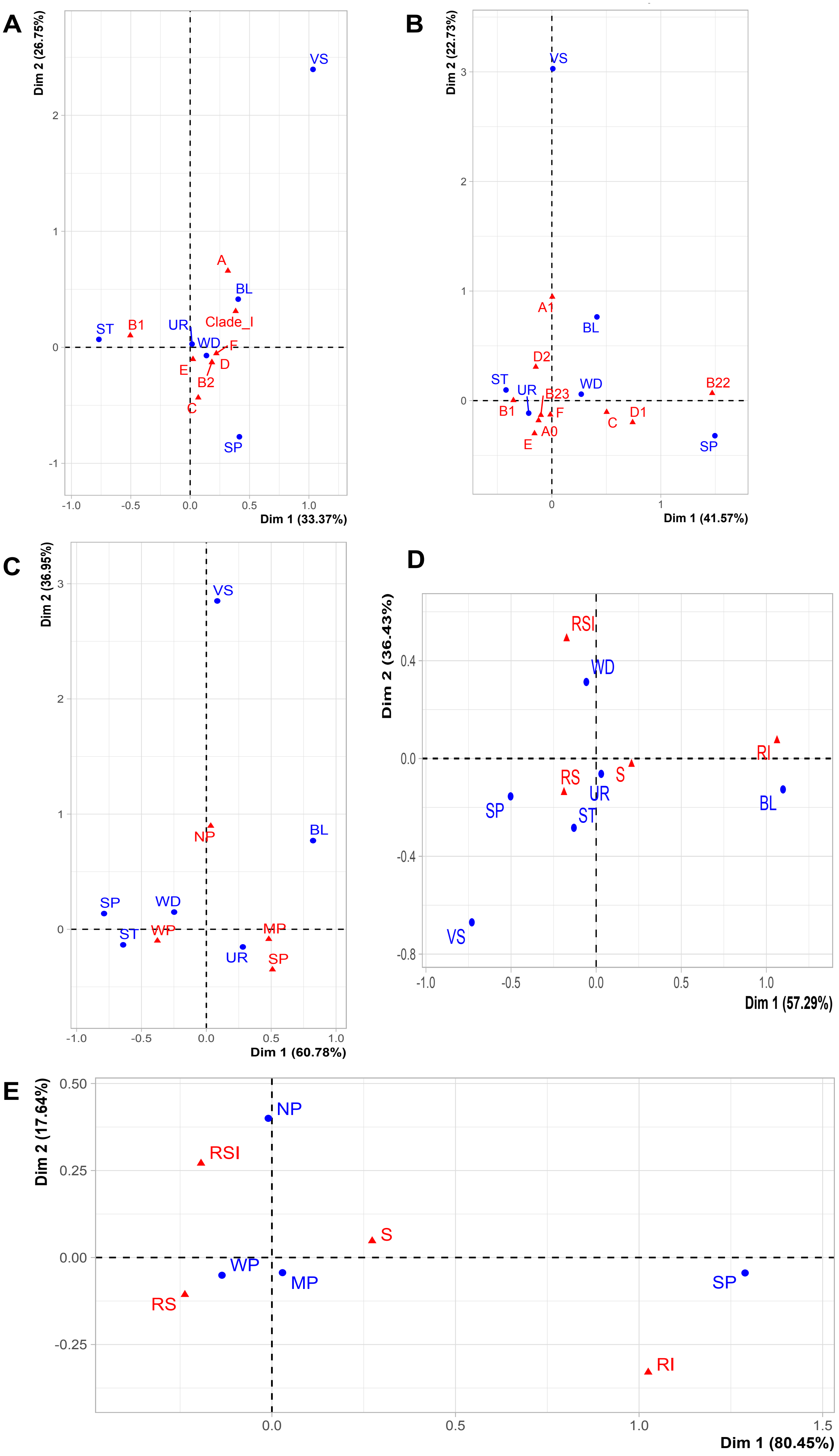
A
Dim 2 (26.75%)
Dim 1 (33.37%)
VS
A
BL
ST
B1
UR
Clade_I
WD
F
E
D
B2
C
SP
B
Dim 2 (22.73%)
Dim 1 (41.57%)
VS
A1
BL
D2
ST
UR
B23
WD
B22
B1
F
C
D1
A0
E
SP
C
Dim 2 (36.95%)
Dim 1 (60.78%)
VS
NP
BL
SP
WD
ST
WP
MP
UR
SP
D
Dim 2 (36.43%)
Dim 1 (57.29%)
RSI
WD
RI
RS
S
UR
SP
ST
BL
VS
E
Dim 2 (17.64%)
Dim 1 (80.45%)
NP
RSI
S
WP
MP
SP
RS
RI

Fig. S2: Correspondence analyses (CA) of categorical variables of *E. coli* isolates: **A.** clinical source of the isolates vs phylotypes; **B.** clinical source of the isolates vs phylotypes (subgroups); **C.** clinical source of the isolates vs biofilm formation capacity; **D.** clinical source of the isolates vs serum resistance; and **E.** biofilm formation capacity vs serum resistance.

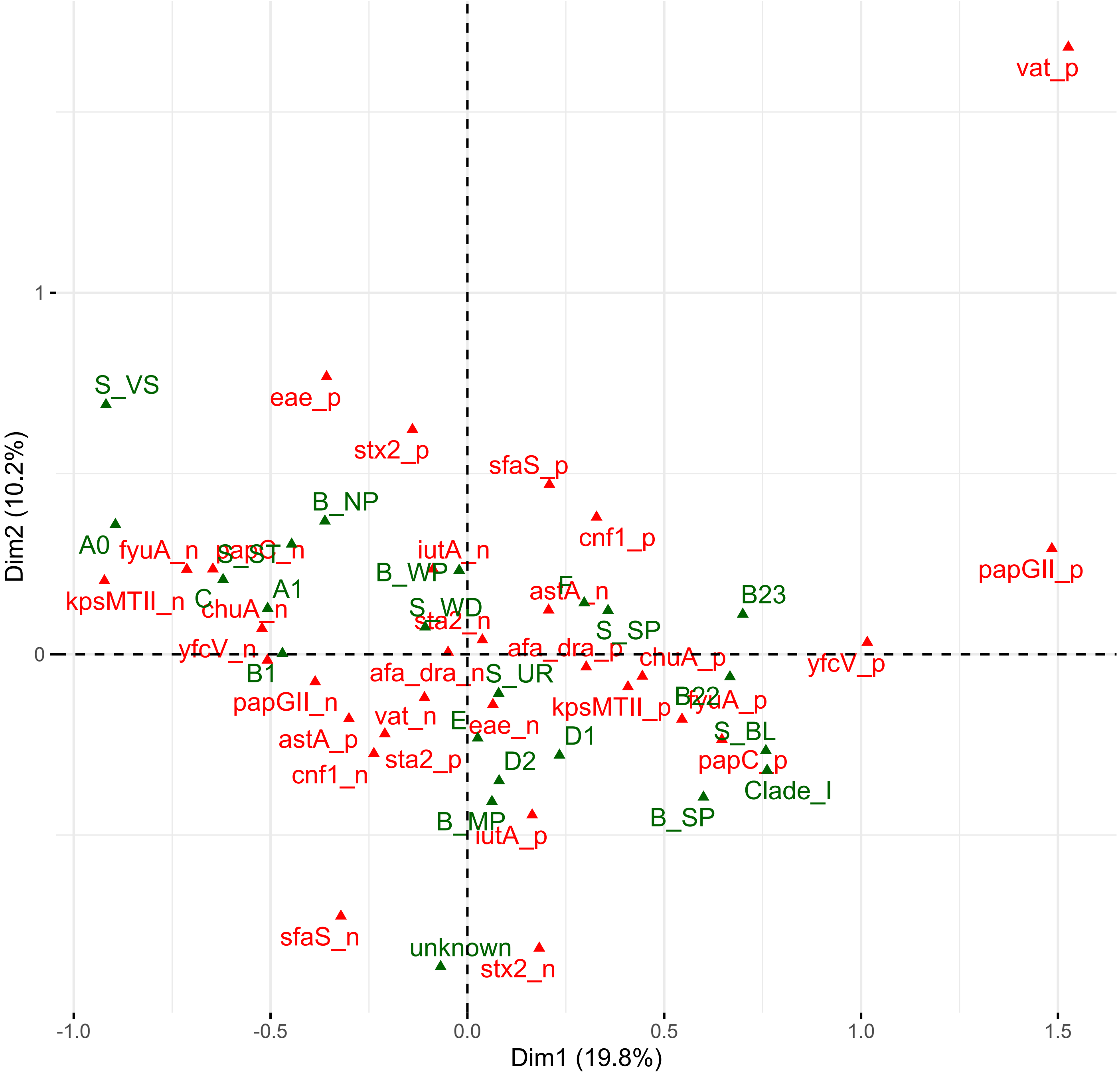

Fig. S3: Multiple correspondence analysis (MCA) of *E. coli* isolates clustered according to virulence encoding genes (active variables); while clinical source of the isolates, phylotypes (subgroups) and biofilm formation capacity were used as supplementary variables.
